# Body Mass Index and Postacute Sequelae of SARS-CoV-2 Infection in Children and Young Adults

**DOI:** 10.1001/jamanetworkopen.2024.41970

**Published:** 2024-10-28

**Authors:** Ting Zhou, Bingyu Zhang, Dazheng Zhang, Qiong Wu, Jiajie Chen, Lu Li, Yiwen Lu, Michael J. Becich, Saul Blecker, Nymisha Chilukuri, Elizabeth A. Chrischilles, Haitao Chu, Leonor Corsino, Carol R. Geary, Mady Hornig, Maxwell M. Hornig-Rohan, Susan Kim, David M. Liebovitz, Vitaly Lorman, Chongliang Luo, Hiroki Morizono, Abu S. M. Mosa, Nathan M. Pajor, Suchitra Rao, Hanieh Razzaghi, Srinivasan Suresh, Yacob G. Tedla, Leah Vance Utset, Youfa Wang, David A. Williams, Margot Gage Witvliet, Caren Mangarelli, Ravi Jhaveri, Christopher B. Forrest, Yong Chen

**Affiliations:** 1 The Center for Health AI and Synthesis of Evidence (CHASE), University of Pennsylvania, Philadelphia; 2 Department of Biostatistics, Epidemiology, and Informatics, University of Pennsylvania Perelman School of Medicine, Philadelphia; 3 The Graduate Group in Applied Mathematics and Computational Science, School of Arts and Sciences, University of Pennsylvania, Philadelphia; 4 Department of Biostatistics and Health Data Science, University of Pittsburgh, Pittsburgh, Pennsylvania; 5 Department of Biomedical Informatics, University of Pittsburgh School of Medicine, Pittsburgh, Pennsylvania; 6 Department of Population Health, New York University Grossman School of Medicine, New York; 7 Department of Pediatrics, Stanford University School of Medicine, Stanford, California; 8 Department of Epidemiology, College of Public Health, The University of Iowa, Iowa City; 9 Division of Biostatistics, University of Minnesota School of Public Health, Minneapolis; 10 Statistical Research and Innovation, Global Biometrics and Data Management, Pfizer Inc, New York, New York; 11 Department of Medicine, Duke University School of Medicine, Durham, North Carolina; 12 Department of Pathology, Microbiology, and Immunology, University of Nebraska Medical Center, Omaha; 13 RECOVER Patient, Caregiver, or Community Advocate Representative, New York, New York; 14 Department of Epidemiology, Columbia University Mailman School of Public Health, New York, New York; 15 Department of Pediatrics, Division of Rheumatology, University of California San Francisco Benioff Children’s Hospital, San Francisco; 16 Division of General Internal Medicine, Feinberg School of Medicine, Northwestern University, Chicago, Illinois; 17 Applied Clinical Research Center, The Children’s Hospital of Philadelphia, Philadelphia, Pennsylvania; 18 Division of Public Health Sciences, Washington University School of Medicine in St Louis, St Louis, Missouri; 19 Center for Genetic Medicine Research, Children’s Research Institute, Children’s National Hospital, Washington DC; 20 Department of Biomedical Informatics, Biostatistics, and Medical Epidemiology, University of Missouri School of Medicine, Columbia; 21 Division of Pulmonary Medicine, Cincinnati Children’s Hospital Medical Center, Cincinnati, Ohio; 22 Department of Pediatrics, University of Cincinnati College of Medicine, Cincinnati, Ohio; 23 Department of Pediatrics, University of Colorado School of Medicine, Aurora; 24 Divisions of Health Informatics & Emergency Medicine, Department of Pediatrics, University of Pittsburgh, Pittsburgh, Pennsylvania; 25 UPMC Children’s Hospital of Pittsburgh, Pittsburgh, Pennsylvania; 26 Division of Epidemiology, Department of Medicine, Vanderbilt University Medical Center, Nashville, Tennessee; 27 Division of Primary Care Pediatrics, Nationwide Children’s Hospital, Columbus, Ohio; 28 Global Health Institute, Xi’an Jiaotong University, Xi’an, China; 29 Department of Anesthesiology, University of Michigan, Ann Arbor; 30 Department of Sociology, Social Work and Criminal Justice, Lamar University, Beaumont, Texas; 31 Division of Advanced General Pediatrics and Primary Care, Ann & Robert H. Lurie Children’s Hospital of Chicago, Chicago, Illinois; 32 Division of Pediatric Infectious Diseases, Ann & Robert H. Lurie Children’s Hospital of Chicago, Chicago, Illinois; 33 Leonard Davis Institute of Health Economics, University of Pennsylvania, Philadelphia; 34 Penn Medicine Center for Evidence-Based Practice (CEP), University of Pennsylvania, Philadelphia; 35 Penn Institute for Biomedical Informatics (IBI), University of Pennsylvania, Philadelphia

## Abstract

**Importance:**

Obesity is associated with increased severity of COVID-19. Whether obesity is associated with an increased risk of post-acute sequelae of SARS-CoV-2 infection (PASC) among pediatric populations, independent of its association with acute infection severity, is unclear.

**Objective:**

To quantify the association of body mass index (BMI) status before SARS-CoV-2 infection with pediatric PASC risk, controlling for acute infection severity.

**Design, Setting, and Participants:**

This retrospective cohort study occurred at 26 US children’s hospitals from March 2020 to May 2023 with a minimum follow-up of 179 days. Eligible participants included children and young adults aged 5 to 20 years with SARS-CoV-2 infection. Data analysis was conducted from October 2023 to January 2024.

**Exposures:**

BMI status assessed within 18 months before infection; the measure closest to the index date was selected. The BMI categories included healthy weight (≥5th to <85th percentile for those aged 5-19 years or ≥18.5 to <25 for those aged >19 years), overweight (≥85th to <95th percentile for those aged 5-19 years or ≥25 to <30 for for those aged >19 years), obesity (≥95th percentile to <120% of the 95th percentile for for those aged 5-19 years or ≥30 to <40 for those aged >19 years), and severe obesity (≥120% of the 95th percentile for those aged 5-19 years or ≥40 for those aged >19 years).

**Main Outcomes And Measures:**

To identify PASC, a diagnostic code specific for post–COVID-19 conditions was used and a second approach used clusters of symptoms and conditions that constitute the PASC phenotype. Relative risk (RR) for the association of BMI with PASC was quantified by Poisson regression models, adjusting for sociodemographic, acute COVID severity, and other clinical factors.

**Results:**

A total of 172 136 participants (mean [SD] age at BMI assessment 12.6 [4.4] years; mean [SD] age at cohort entry, 13.1 [4.4] years; 90 187 female [52.4%]) were included. Compared with participants with healthy weight, those with obesity had a 25.4% increased risk of PASC (RR, 1.25; 95% CI, 1.06-1.48) and those with severe obesity had a 42.1% increased risk of PASC (RR, 1.42; 95% CI, 1.25-1.61) when identified using the diagnostic code. Compared with those with healthy weight, there was an increased risk for any occurrences of PASC symptoms and conditions among those with obesity (RR, 1.11; 95% CI, 1.06-1.15) and severe obesity (RR, 1.17; 95% CI, 1.14-1.21), and the association held when assessing total incident occurrences among those with overweight (RR, 1.05; 95% CI, 1.00-1.11), obesity (RR, 1.13; 95% CI, 1.09-1.19), and severe obesity (RR, 1.18; 95% CI, 1.14-1.22).

**Conclusions And Relevance:**

In this cohort study, elevated BMI was associated with a significantly increased PASC risk in a dose-dependent manner, highlighting the need for targeted care to prevent chronic conditions in at-risk children and young adults.

## Key Points

**Question:**

Is elevated body mass index (BMI) associated with an increased risk of developing post-acute sequelae of SARS-Cov-2 infection (PASC) among children and young adults?

**Findings:**

In this cohort study of 172 136 children and young adults, elevated BMI was associated with significantly higher risk of PASC. Compared with peers with a healthy BMI, there was a 25.4% increased risk of PASC among those with obesity and a 42.1% increased risk of PASC among those with severe obesity.

**Meaning:**

These findings suggest that overweight and obesity are important risk factors for pediatric PASC, highlighting the need for targeted care to prevent chronic conditions in at-risk children and young adults who are infected with SARS-CoV-2.

## Teaser

This cohort study analyzes associations of body mass index (BMI) with development of postacute sequalae of SARS-CoV-2 among children and young adults.

## Introduction

Postacute sequelae of SARS-CoV-2 infection (PASC) encompasses a broad and heterogeneous array of persistent, relapsing, or newly emerging symptoms persisting beyond at least 4 weeks after the acute phase of COVID-19.^1,2,3,4^ This condition exhibits multifaceted involvement across various organ systems.^5,6,7,8^ The prevalence of pediatric PASC with SARS-CoV-2 infection varies across studies, with reported rates ranging from 1.6% to 70%.^9,10,11,12^ PASC continues to pose a substantial threat to children, necessitating an urgent and deeper understanding of pediatric PASC causes. This research priority is underscored in the updated National Institute for Health and Care Excellence guideline.^13^ This need to unravel the complexities of pediatric PASC continues to be crucial even after the COVID-19 pandemic.

Obesity is now one of the most common chronic diseases in the US, impacting more than 40% of adults and approximately 20% of children.^14,15^ The association of obesity with severe adverse outcomes was seen again with the onset of the COVID-19 pandemic.^16^ The association of obesity with increased risk of PASC has been widely discussed, with a predominant focus on adults.^17,18^ Some studies have reported that overweight or obesity is associated with an elevated risk of PASC^18,19^ across different timelines, irrespective of whether the definition of PASC extends to 12 weeks^18^ or 4 months.^19^ However, the nuances of this association become apparent when considering the specific timeline for defining PASC. For example, Sudre et al^20^ observed that increasing body mass index (BMI) and obesity were associated with higher odds of PASC lasting for more than 4 weeks, but this association did not extend to PASC lasting for more than 12 weeks.

While some studies^21,22,23^ have examined PASC in pediatric populations, including obesity as a risk factor, the association of BMI with PASC among children remains less studied than in adults, especially regarding the potential dose-response association. This gap in research is crucial, giving potential long-term implications for children’s health. Our study addresses this gap by analyzing data from 26 US children’s hospitals or institutions, exploring the association of preinfection BMI with PASC outcomes. The findings may help inform preventive strategies and clinical management for at-risk pediatric patients.

## Methods

### Data Sources

This retrospective cohort study was approved with a waiver of informed consent due to the use of deidentified data by the Biomedical Research Alliance of New York institutional review board and follows the Strengthening the Reporting of Observational Studies in Epidemiology (STROBE) reporting guideline.^24^ This study is part of the National Institutes of Health Researching COVID-19 to Enhance Recovery, which aims to understand, treat, and prevent PASC.^25^ Twenty-six institutions contributed to the data, including data from both hospitals and primary care or outpatient settings, ensuring a comprehensive analysis of pediatric COVID-19 outcomes (see eTable 1 in Supplement 1 for details).

This study derived electronic health record (EHR) data from March 2020 to May 2023 by including patients with a documented SARS-CoV-2 infection younger than 21 years, aligning with the American Academy of Pediatrics definition of the pediatric population,^26^ who had at least 1 visit within the baseline period of 18 months to 7 days prior to the index date and had at least 1 visit within the follow-up period of 28 days to 179 days after the index date. Documented SARS-CoV-2 infections were defined by SARS-CoV-2 polymerase chain reaction testing; antigen or serology positive testing; or diagnosis of COVID-19, PASC, or multisystem inflammatory syndrome. The index date was set as either the earliest date of positive tests or COVID-19 diagnoses or 28 days before PASC or multisystem inflammatory syndrome diagnosis. Data are described in the eMethods in Supplement 1.

Participants were excluded if they were younger than 5 years at the time of assessing BMI, due to their potential for more dramatic BMI variations during the baseline period, and if they had genetic syndromes associated with obesity or any conditions signaling a need for weight gain or a medical cause of altered weight tendencies during the baseline period (eTable 2 in Supplement 1). The participants selection process is summarized in the eFigure in Supplement 1.

### Defining BMI Status

When multiple BMI measures (calculated as weight in kilograms divided by height in meters squared) were available during the baseline period, we selected the measure closest to the index date. For participants aged 5 to 19 years, according to the age- and sex-specific BMI percentiles based on US Centers for Disease Control and Prevention growth charts, the BMI status was categorized into healthy weight (5th percentile to less than 85th percentile), overweight (85th percentile to less than 95th percentile), obesity (95th percentile to less than 120% of the 95th percentile), and severe obesity (120% of the 95th percentile or greater)^27^; for participants older than 19 years, these 4 categories were divided by BMI of 18.5 to less than 25, 25 to less than 30, 30 to less than 40, and 40 or more within the baseline period.^28^

### Defining PASC

The outcome was assessed within the follow-up period. We first used* International Statistical Classification of Diseases, Tenth Revision, Clinical Modification (ICD-10-CM) *code U09.9, specific for post–COVID-19 condition, to indicate PASC. For this, we created a binary outcome, PASC (U09.9), which was defined as positive if anyone was identified as having PASC with a U09.9 diagnosis code.

Second, we used clusters of symptoms and health conditions previously shown to constitute the PASC phenotype based on experts’ suggestions,^23^ including abdominal pain, abnormal liver enzyme, acute kidney injury, acute respiratory distress syndrome, arrhythmias, cardiovascular signs and symptoms, changes in taste and smell, chest pain, cognitive dysfunction, fatigue and malaise, fever and chills, fluid and electrolyte imbalances, generalized pain, hair loss, headache, heart disease, mental health disorders, musculoskeletal pain, myocarditis, myositis, postural orthostatic tachycardia syndrome or dysautonomia, respiratory signs and symptoms, skin symptoms, thrombophlebitis, and thromboembolism. We assessed incident occurrences of these 24 PASC symptoms and conditions that occurred within the follow-up period but did not occur during the baseline period, and then a binary and a count outcome were created. The binary outcome was defined as positive if any of these conditions occurred, and the count outcome was defined as total incident occurrences of these conditions.

### Covariates

Age at BMI status assessment and age at cohort entry were collected. Other demographic characteristics included sex (male or female), and EHR-derived race and ethnicity (Asian American or Pacific Islander, Hispanic, non-Hispanic Black, non-Hispanic White). The predominating COVID-19 virus variant was categorized as pre-Alpha (March 1, 2020, to March 31, 2021), Alpha (April 1, 2021, to June 30, 2021), Delta (July 1, 2021, to December 31, 2021), and Omicron (January 1, 2022, to December 1, 2022).^29^ We used numbers of emergency department visits, outpatient department visits, inpatient department visits, medications or prescriptions, and negative COVID-19 tests up to 24 months before index date as health care utilization metrics, and the pediatric medical complexity algorithm (PMCA)^30^ index to address levels of medical complexity comorbidity (no condition, noncomplex condition, or complex chronic condition) for the participants. We also classified participants based on the acute COVID-19 severity as asymptomatic, mild, moderate, or severe.^31^ Doses of COVID-19 vaccine prior to infection and interval since the last COVID-19 vaccination date (no vaccine, <4 months, and ≥4 months), and type of insurance (private, public, or other) were also considered in the secondary analyses.

### Statistical Analysis

#### Primary Analysis

We first presented the demographic and clinical characteristics across different BMI statuses. Comparisons of PASC (U09.9) and potential PASC symptoms and conditions were then made by BMI status. For the count outcome, total incident occurrences of PASC symptoms and conditions, we used Poisson regression to assess its association with BMI status by estimating the relative risk (RR) and 95% CI, adjusting for demographic characteristics (age, sex, and race and ethnicity), predominant variant, health care utilization metrics prior to cohort entry, PMCA index, and acute COVID-19. For binary outcomes, PASC (U09.9) and any incident occurrences of PASC symptoms and conditions, modified Poisson regression^32^ was employed, accounting for possible variance overestimation and adjusting for the factors described previously. Orthogonal trend analysis was used for the linear trend test. Statistical methods are described in eAppendix in Supplement 1. All analyses were conducted using R version 4.1.2 (R Project for Statistical Computing). Statistical significance was set at *P* < .05 (2-tailed). Data analysis was conducted from October 2023 to January 2024.

#### Post Hoc Secondary Analysis

We conducted exploratory subgroup analyses based on age (<18 or ≥18 years) and PMCA index (no chronic condition vs noncomplex or complex chronic condition), and race and ethnicity, acknowledging disparities in children and young adult, the presence or severity of chronic conditions, and BMI status and COVID-19 severity by race and ethnicity to explore potential effect modification.

To validate our findings, we performed several post hoc sensitivity analyses. These included excluding participants with BMI assessed more than 6 months before cohort entry because pediatric BMI increased during the pandemic;^33^ those entering the study before the *ICD-10-CM* code U09.9 became effective on October 1, 2021; those who were included with the diagnosis of PASC alone; and those diagnosed solely by serology testing after November 2022 due to potential accuracy concerns during the Omicron wave.^34,35^ We also excluded participants with moderate or severe infection, those with diabetes,^36,37^ and those without diabetes but using weight-loss drugs. Additionally, we adjusted for vaccination status before infection,^38,39^ insurance type, and site index. Lastly, we assessed our findings in a primary care setting and used a negative control outcome (foreign body in ear) to evaluate residual bias.^40,41^

## Results

After excluding 221 participants who had underweight and 67 451 with missing BMI status, a total of 172 136 participants (mean [SD] age at BMI assessment 12.6 [4.4] years; mean [SD] age at cohort entry, 13.1 [4.4] years; 90 187 female [52.4%]; 42 982 Hispanic [25.0]; 33 065 non-Hispanic Black [19.2%]; 87 275 non-Hispanic White [50.7%]) were included in the analysis. Participants missing BMI status data were more likely to be younger, be male, be non-Hispanic Black or non-Hispanic White, be infected after the pre-Alpha wave, have fewer complex chronic conditions, utilize health care less, and were less likely to develop PASC (eTable 3 in Supplement 1). The median (IQR) time from BMI status assessment to COVID-19 infection was 4.1 (1.6-8.6) months. Of all the participants, 85 613 (49.7%) had obesity or severe obesity (Table 1).

**Table 1.** Characteristics of Pediatric Participants by BMI Status Prior to the SARS-CoV-2 Infection^a^

Characteristics	Participants by BMI status, No. (%)				
	Healthy weight (n = 68 918)	Overweight (n = 17 605)	Obesity (n = 25 372)	Severe obesity (n = 60 241)	Overall (N = 172 136)
Age, mean (SD), y					
Assessed BMI	12.6 (4.5)	12.8 (4.3)	11.3 (4.2)	13.1 (4.2)	12.6 (4.4)
Entered cohort	13.0 (4.5)	13.2 (4.3)	11.8 (4.2)	13.6 (4.2)	13.1 (4.4)
Sex					
Female	36 011 (52.3)	9580 (54.4)	11 627 (45.8)	32 969 (54.7)	90 187 (52.4)
Male	32 907 (47.7)	8025 (45.6)	13 745 (54.2)	27 272 (45.3)	81 949 (47.6)
Race and ethnicity					
Asian American or Pacific Islander	3996 (5.8)	770 (4.4)	1055 (4.2)	2993 (5.0)	8814 (5.1)
Hispanic	10 657 (15.5)	3588 (20.4)	7335 (28.9)	21 402 (35.5)	42 982 (25.0)
Non-Hispanic Black	11 980 (17.4)	3854 (21.9)	6004 (23.7)	11 227 (18.6)	33 065 (19.2)
Non-Hispanic White	42 285 (61.4)	9393 (53.4)	10 978 (43.3)	24 619 (40.9)	87 275 (50.7)
Predominant variant					
Pre-Alpha	18 204 (26.4)	4592 (26.1)	5920 (23.3)	16 724 (27.8)	45 440 (26.4)
Alpha	3641 (5.3)	968 (5.5)	1354 (5.3)	2592 (4.3)	8555 (5.0)
Delta	17 001 (24.7)	4688 (26.6)	7063 (27.8)	13 738 (22.8)	42 490 (24.7)
Omicron	30 072 (43.6)	7357 (41.8)	11 035 (43.5)	27 187 (45.1)	75 651 (43.9)
Pediatric medical complexity algorithm					
None	41 656 (60.4)	10 071 (57.2)	14 427 (56.9)	39 140 (65.0)	105 294 (61.2)
Noncomplex	15 755 (22.9)	4470 (25.4)	6521 (25.7)	13 389 (22.2)	40 135 (23.3)
Complex	11 507 (16.7)	3064 (17.4)	4424 (17.4)	7712 (12.8)	26 707 (15.5)
Acute COVID-19 severity					
Asymptomatic	43 807 (63.6)	11 277 (64.1)	15 528 (61.2)	31 754 (52.7)	102 366 (59.5)
Mild	20 867 (30.3)	5205 (29.6)	8309 (32.7)	26 347 (43.7)	60 728 (35.3)
Moderate	2617 (3.8)	725 (4.1)	978 (3.9)	1384 (2.3)	5704 (3.3)
Severe	1627 (2.4)	398 (2.3)	557 (2.2)	756 (1.3)	3338 (1.9)
Location of diagnosed COVID-19					
ED	7830 (11.4)	2562 (14.6)	3511 (13.8)	3185 (5.3)	17 088 (9.9)
IPD	2325 (3.4)	548 (3.1)	764 (3.0)	934 (1.6)	4 71 (2.7)
OPD	28 645 (41.6)	6898 (39.2)	12 126 (47.8)	41 270 (68.5)	88 939 (51.7)
OPD: test only	13 700 (19.9)	3623 (20.6)	4814 (19.0)	9750 (16.2)	31 887 (18.5)
Other or unknown	16 418 (23.8)	3974 (22.6)	4157 (16.4)	5102 (8.5)	29 651 (17.2)
No. of negative COVID-19 tests					
0	39 837 (57.8)	10 073 (57.2)	14 939 (58.9)	39 463 (65.5)	104 312 (60.6)
1	15 426 (22.4)	3990 (22.7)	5671 (22.4)	11 877 (19.7)	36 964 (21.5)
≥2	13 655 (19.8)	3542 (20.1)	4762 (18.8)	8901 (14.8)	30 860 (17.9)
No. of ED visits					
0	49 015 (71.1)	11 729 (66.6)	17 601 (69.4)	51 517 (85.5)	129 862 (75.4)
1	11 041 (16.0)	3021 (17.2)	3868 (15.2)	4966 (8.2)	22 896 (13.3)
2	4368 (6.3)	1270 (7.2)	1682 (6.6)	1798 (3.0)	9118 (5.3)
≥3	4494 (6.5)	1585 (9.0)	2221 (8.8)	1960 (3.3)	10 260 (6.0)
No. of IPD visits					
0	62 276 (90.4)	15 831 (89.9)	23 123 (91.1)	57 596 (95.6)	158 826 (92.3)
1	4101 (6.0)	1132 (6.4)	1462 (5.8)	1709 (2.8)	8404 (4.9)
2	1191 (1.7)	306 (1.7)	400 (1.6)	486 (0.8)	2383 (1.4)
≥3	1350 (2.0)	336 (1.9)	387 (1.5)	450 (0.7)	2523 (1.5)
No. of OPD visits					
0	2315 (3.4)	643 (3.7)	1374 (5.4)	4498 (7.5)	8830 (5.1)
1	6570 (9.5)	1751 (9.9)	2409 (9.5)	4544 (7.5)	15 274 (8.9)
2	8355 (12.1)	2105 (12.0)	2866 (11.3)	5929 (9.8)	19 255 (11.2)
≥3	51 678 (75.0)	13 106 (74.4)	18 723 (73.8)	45 270 (75.1)	128 777 (74.8)
No. of medications or prescriptions					
0	9944 (14.4)	2235 (12.7)	3878 (15.3)	12 364 (20.5)	28 421 (16.5)
1	7759 (11.3)	1787 (10.2)	2627 (10.4)	7078 (11.7)	19 251 (11.2)
2	6998 (10.2)	1639 (9.3)	2404 (9.5)	6591 (10.9)	17 632 (10.2)
≥2	44 217 (64.2)	11 944 (67.8)	16 463 (64.9)	34 208 (56.8)	106 832 (62.1)
Vaccine doses, No.					
0	53 946 (78.3)	13 975 (79.4)	20 914 (82.4)	46 248 (76.8)	135 083 (78.5)
1	2519 (3.7)	620 (3.5)	901 (3.6)	2576 (4.3)	6616 (3.8)
2	9979 (14.5)	2477 (14.1)	3065 (12.1)	9308 (15.5)	24 829 (14.4)
≥3	2474 (3.6)	533 (3.0)	492 (1.9)	2109 (3.5)	5608 (3.3)

Abbreviations: BMI, body mass index; ED, emergency department; IPD, inpatient department; OPD, outpatient department.

^a^ Percentages may not total 100 because of rounding.

During the follow-up period, 1402 participants (0.8%) received a diagnosis of PASC (U09.9), of which 751 (53.6%) had obesity or severe obesity, and 63 046 (26.4%) had at least 1 incident occurrence of PASC symptoms and conditions, of which 8792 (52.8%) had obesity or severe obesity. The median (IQR) time from index date to outcomes (ie, the diagnosis of U09.9 and the first incident occurrence of any of the 24 PASC symptoms and conditions) was 50 (28-111) days and 63 (38-105) days, respectively. The median (IQR) total number of incident occurrences of PASC symptoms and conditions was 0 (0-2), signifying that at least one-half of the participants in the cohort did not experience any incident occurrences of PASC symptoms and conditions. Incident occurrences of each PASC symptoms and conditions are in eTable 4 in Supplement 1.

Participants with overweight, obesity, or severe obesity exhibited an increased risk of PASC compared with those with healthy weight; however, not all estimates reached statistical significance. Specifically, in comparison with those with healthy weight, there was a higher risk of PASC (U09.9) among participants categorized as having obesity (RR, 1.25; 95% CI, 1.06-1.48) and severe obesity (RR, 1.42; 95% CI, 1.25-1.61). Similarly, compared with those with healthy weight, there was an increased likelihood of encountering any manifestation of potential PASC symptoms and conditions among those who had obesity (RR, 1.11; 95% CI, 1.06-1.15) and severe obesity (RR, 1.17; 95% CI, 1.14-1.21). The association became slightly more pronounced when assessing the cumulative occurrences of PASC symptoms and conditions among those who had overweight (RR, 1.05; 95% CI, 1.00-1.11), obesity (RR, 1.14; 95% CI, 1.09-1.19), and severe obesity (RR, 1.18; 95% CI, 1.14-1.22). There was a significant dose-response association of increasing BMI category with risk of PASC (Table 2).

**Table 2.** BMI Status Prior to SARS-CoV-2 Infection and Risk of PASC^a^

Outcome by BMI status	Incident vs total COVID-19 cases, No./total No. (%)^b^	RR (95% CI)	*P* value for trend
PASC^c^			
Healthy weight	514/68 918 (0.7)	1 [Reference]	.001
Overweight	137/17 605 (0.8)	1.05 (0.87-1.26)	
Obesity	199/25 372 (0.8)	1.25 (1.06-1.48)	
Severe obesity	552/60 241 (0.9)	1.42 (1.25-1.61)	
PASC symptoms and conditions			
Any occurrences			
Healthy weight	28 674/68 918 (41.6)	1 [Reference]	<.001
Overweight	7637/17 605 (43.4)	1.03 (0.98-1.08)	
Obesity	11 081/25 372 (43.7)	1.11 (1.06-1.15)	
Severe obesity	26 925/60 241 (44.7)	1.17 (1.14-1.21)	
Total occurrences, median (IQR)			
Healthy weight	0 (0-2)	1 [Reference]	<.001
Overweight	0 (0-2)	1.05 (1.00-1.11)	
Obesity	0 (0-2)	1.14 (1.09-1.19)	
Severe obesity	0 (0-2)	1.18 (1.14-1.22)	

Abbreviations: BMI, body mass index; PASC, post-acute sequelae of SARS-CoV-2 infection; RR, relative risk.

^a^ Adjusted for age at BMI assessment and at cohort entry (continuous), sex, race and ethnicity, pediatric medical complexity algorithm index, predominant variant, acute COVID-19 severity, numbers of emergency department visits, outpatient department visits, inpatient department visits, medications or prescriptions, and negative COVID-19 tests.

^b^ Incident refers to the count of participants who developed the outcome we were interested in (ie, PASC or PASC symptoms and conditions); total COVID-19 cases refers to the count of the participants in the corresponding group; and the value in the parentheses refers to the percentage of the groups who developed the outcome.

^c^ *International Statistical Classification of Diseases, Tenth Revision, Clinical Modification (ICD-10-CM) *code U09.9.

Subgroup analyses by age and PMCA index showed associations like the main findings. Post hoc analysis of racial and ethnic subgroups revealed a consistent association of BMI with PASC (U09.9) or PASC symptoms for non-Hispanic White participants. However, no significant association of BMI with PASC (U09.9) was observed among non-Hispanic Black participants, nor for any PASC outcomes among Hispanic participants (Table 3). Analysis for Asian American and Pacific Islander participants was not conducted due to insufficient U09.9 diagnoses across BMI categories. Post hoc sensitivity analysis generally supported the dose-response association, with slight variations in the associations (eTables 5-17 in Supplement 1). Negative control outcome analysis showed no significant results, suggesting absence of residual bias (eTable 18 and eTable 19 in Supplement 1).

**Table 3.** BMI Status Prior to SARS-CoV-2 Infection and Risk of PASC in Subgroups Based on Age, PMCA Index, and Race and Ethnicity

Outcome by characteristic and BMI status	Incident vs total COVID-19 cases, No./total No. (%)^a^	RR (95% CI)	*P* value for trend
Age <18 y (n = 145 975)^b^^,^^c^			
PASC^d^			
Healthy weight	408/57 916 (0.7)	1 [Reference]	.008
Overweight	110/14 994 (0.7)	1.05 (0.85-1.30)	
Obesity	173/23 198 (0.7)	1.28 (1.07-1.53)	
Severe obesity	424/49 867 (0.9)	1.43 (1.24-1.65)	
PASC symptoms and conditions			
Any occurrences			
Healthy weight	23 860/57 916 (41.2)	1 [Reference]	<.001
Overweight	6409/14 994 (42.7)	1.02 (0.97-1.07)	
Obesity	9991/23 198 (43.1)	1.10 (1.05-1.15)	
Severe obesity	21 923/49 867 (44.0)	1.17 (1.13-1.21)	
Total occurrences, median (IQR)			
Healthy weight	0 (0-2)	1 [Reference]	<.001
Overweight	0 (0-2)	1.04 (0.99-1.10)	
Obesity	0 (0-2)	1.12 (1.07-1.17)	
Severe obesity	0 (0-2)	1.18 (1.14-1.23)	
Age ≥18 y (n = 26 161)^b^^,^^c^			
PASC^d^			
Healthy weight	106/11 002 (1.0)	1 [Reference]	.049
Overweight	27/2611 (1.0)	1.01 (0.66-1.55)	
Obesity	26/2174 (1.2)	1.14 (0.74-1.76)	
Severe obesity	128/10 374 (1.2)	1.37 (1.05-1.78)	
PASC symptoms and conditions			
Any occurrences			
Healthy weight	4814/11 002 (43.8)	1 [Reference]	<.001
Overweight	1228/2611 (47.0)	1.10 (0.97-1.25)	
Obesity	1090/2174 (50.1)	1.20 (1.05-1.37)	
Severe obesity	5002/10 374 (48.2)	1.20 (1.11-1.30)	
Total occurrences, median (IQR)			
Healthy weight	0 (0-2)	1 [Reference]	.002
Overweight	0 (0-2)	1.14 (0.99-1.32)	
Obesity	2 (0-2)	1.30 (1.12-1.51)	
Severe obesity	0 (0-2)	1.19 (1.09-1.30)	
PMCA index, no chronic condition (n = 105 294)^e^			
PASC^d^			
Healthy weight	316/41 656 (0.8)	1 [Reference]	.02
Overweight	73/10 071 (0.7)	0.97 (0.75-1.24)	
Obesity	112/14 427 (0.8)	1.26 (1.02-1.57)	
Severe obesity	353/39 140 (0.9)	1.46 (1.24-1.71)	
PASC symptoms and conditions			
Any occurrences			
Healthy weight	14 914/41 656 (35.8)	1 [Reference]	<.001
Overweight	3718/10 071 (36.9)	1.00 (0.94-1.06)	
Obesity	5423/14 427 (37.6)	1.12 (1.06-1.17)	
Severe obesity	15 488/39 140 (39.6)	1.18 (1.14-1.23)	
Total occurrences, median (IQR)			
Healthy weight	0 (0-2)	1 [Reference]	<.001
Overweight	0 (0-2)	1.02 (0.96-1.09)	
Obesity	0 (0-2)	1.14 (1.09-1.21)	
Severe obesity	0 (0-2)	1.20 (1.15-1.24)	
PMCA index, noncomplex or complex chronic condition (n = 66 842)^e^			
PASC (U09.9)^d^			
Healthy weight	198/27 262 (0.7)	1 [Reference]	.01
Overweight	64/7534 (0.8)	1.17 (0.88-1.55)	
Obesity	87/10 945 (0.8)	1.26 (0.98-1.62)	
Severe obesity	199/21 101 (0.9)	1.39 (1.14-1.71)	
PASC symptoms and conditions			
Any occurrences			
Healthy weight	13 760/27 262 (50.5)	1 [Reference]	<.001
Overweight	3919/7534 (52.0)	1.11 (1.02-1.21)	
Obesity	5658/10 945 (51.7)	1.09 (1.01-1.18)	
Severe obesity	11 437/21 101 (54.2)	1.16 (1.09-1.24)	
Total occurrences, median (IQR)			
Healthy weight	2 (0-2)	1 [Reference]	<.001
Overweight	2 (0-2)	1.13 (1.03-1.25)	
Obesity	2 (0-2)	1.12 (1.03-1.22)	
Severe obesity	2 (0-2)	1.16 (1.08-1.24)	
Race and ethnicity, Hispanic (n = 42 982)^f^			
PASC^d^			
Healthy weight	70/10 657 (0.7)	1 [Reference]	.45
Overweight	27/3588 (0.8)	1.11 (0.71-1.73)	
Obesity	48/7335 (0.7)	1.14 (0.79-1.65)	
Severe obesity	129/21 402 (0.6)	1.02 (0.76-1.37)	
PASC symptoms and conditions			
Any occurrences			
Healthy weight	4758/10 657 (44.6)	1 [Reference]	.68
Overweight	1634/3588 (45.5)	1.09 (0.98-1.21)	
Obesity	3184/7335 (43.4)	1.16 (1.07-1.25)	
Severe obesity	8591/21 402 (40.1)	1.15 (1.08-1.23)	
Total occurrences, median (IQR)			
Healthy weight	0 (0-2)	1 [Reference]	.64
Overweight	0 (0-2)	1.13 (1.01-1.26)	
Obesity	0 (0-2)	1.18 (1.08-1.29)	
Severe obesity	0 (0-2)	1.16 (1.08-1.25)	
Race and ethnicity, non-Hispanic Black (n = 33 065)^f^			
PASC^d^			
Healthy weight	53/11 980 (0.4)	1 [Reference]	.20
Overweight	21/3854 (0.5)	1.21 (0.73-2.01)	
Obesity	23/6004 (0.4)	0.92 (0.56-1.49)	
Severe obesity	66/11 227 (0.6)	1.33 (0.91-1.94)	
PASC symptoms and conditions			
Any occurrences			
Healthy weight	4665/11 980 (38.9)	1 [Reference]	<.001
Overweight	1514/3854 (39.3)	1.02 (0.92-1.13)	
Obesity	2370/6004 (39.5)	1.05 (0.96-1.14)	
Severe obesity	4827/11 227 (43.0)	1.15 (1.07-1.24)	
Total occurrences, median (IQR)			
Healthy weight	0 (0-2)	1 [Reference]	<.001
Overweight	0 (0-2)	1.02 (0.91-1.14)	
Obesity	0 (0-2)	1.08 (0.98-1.18)	
Severe obesity	0 (0-2)	1.17 (1.08-1.26)	
Race and ethnicity, non-Hispanic White (n = 87 275)^f^			
PASC^d^			
Healthy weight	371/42 285 (0.9)	1 [Reference]	<.001
Overweight	83/9393 (0.9)	0.96 (0.76-1.22)	
Obesity	120/10 978 (1.1)	1.30 (1.06-1.59)	
Severe obesity	333/24 619 (1.4)	1.55 (1.33-1.80)	
PASC symptoms and conditions			
Any occurrences			
Healthy weight	17 827/42 285 (42.2)	1 [Reference]	<.001
Overweight	4208/9393 (44.8)	1.02 (0.96-1.09)	
Obesity	5090/10 978 (46.4)	1.07 (1.01-1.14)	
Severe obesity	12 363/24 619 (50.2)	1.22 (1.17-1.28)	
Total occurrences, median (IQR)			
Healthy weight	0 (0-2)	1 [Reference]	<.001
Overweight	0 (0-2)	1.04 (0.97-1.12)	
Obesity	0 (0-2)	1.11 (1.03-1.18)	
Severe obesity	2 (0-2)	1.23 (1.17-1.29)	

Abbreviations: BMI, body mass index; PASC, post-acute sequelae of SARS-CoV-2 infection; PMCA, pediatric medical complexity algorithm; RR, relative risk.

^a^ Incident refers to the count of participants who developed the outcome we were interested in (ie, PASC or PASC symptoms and conditions); total COVID-19 cases refers to the count of the participants in the corresponding group; and the value in the parentheses refers to the percentage of the groups who developed the outcome.

^b^ Adjusted for age at BMI assessment and cohort entry (continuous), sex, race and ethnicity, PMCA index, predominant variant, acute COVID-19 severity, number of emergency department visits, outpatient department visits, inpatient department visits, medications or prescriptions, and negative COVID-19 tests.

^c^ Stratified by the cohort entry age.

^d^ *International Statistical Classification of Diseases, Tenth Revision, Clinical Modification (ICD-10-CM) *code U09.9.

^e^ Adjusted for age at BMI assessment and cohort entry (continuous), sex, predominant variant, acute COVID-19 severity, number of emergency department visits, outpatient department visits, inpatient department visits, medications or prescriptions, and negative COVID-19 tests.

^f^ Adjusted for age at BMI assessment and cohort entry (continuous), sex, PMCA index, predominant variant, acute COVID-19 severity, number of emergency department visits, outpatient department visits, inpatient department visits, medications or prescriptions, and negative COVID-19 tests.

## Discussion

To our knowledge, this retrospective cohort study is the first and the largest to explore the association of BMI status with PASC among the pediatric population. Our findings consistently reveal an adverse dose-response association of preinfection BMI with PASC susceptibility, even after adjusting for sociodemographic and clinical factors. This pattern mirrors findings in adult populations,^19^ although the magnitudes of association vary. In exploratory secondary analyses, the association generally remained robust. However, post hoc subgroup analyses by race and ethnicity suggested potential effect size modification, warranting further investigation. An exploratory negative control outcome analysis revealed no substantial residual bias, lending additional support to the primary findings. These post hoc results, while intriguing, should be interpreted cautiously and considered hypothesis-generating for future research.

This research is important because it addresses critical gaps in understanding the long-term impacts of PASC in children, a demographic often overlooked in earlier studies. By identifying the association of elevated BMI with PASC, this study highlights potential risks for prolonged health issues in pediatric populations. The findings suggest that PASC may lead to poorer long-term quality of life, affecting physical health, educational achievement, and social development; this underscores the importance of early identification, prevention, and targeted interventions to mitigate these risks. The implications of this research are crucial for guiding public health strategies and informing clinical practices aimed at improving the long-term well-being of children affected by COVID-19.

A pivotal strength of our study lies in its extensive and representative sample. Covering 172 136 pediatric patients with COVID-19 infection from 26 US children’s hospitals, our study is distinguished by its scale, enabling us ample statistical power to rigorously evaluate the association of BMI with PASC, accounting for key sociodemographic and clinical risk factors. A noteworthy distinction from the previous research is our *ICD-10-CM* coded EHR phenotypes rather than self-reported PASC symptoms because the latter may be influenced by individual perceptions, interpretations, recall biases, lack of standardization, and participant compliance with reporting protocols. The inclusion of multiple PASC outcomes enhances the clarity in understanding the burden and risk of PASC and mitigates the potential for misclassification; this is particularly relevant given the recognized limitations associated with only using *ICD-10-CM* code U09.9^42^ and acknowledging the likely divergence in clinical features of PASC between pediatric and adult populations.^23^ Moreover, a considerable portion of the existing studies primarily focused solely on hospitalized cohorts, while our cohort is characterized by its inclusivity, incorporating both outpatient and hospitalized participants.

Our study revealed a higher obesity rate (35.7%-49.7%) compared with the national average (19.7%),^15^ although rates from primary care sites were comparable (17.5%-22.4%). This disparity may reflect better health among primary care participants and increased health risks among those with overweight or obesity.^43^ Higher BMI has been associated with increased risk of hospitalization^44^ and severe illness^45^ in pediatric patients with COVID-19. Pandemic-related increases in pediatric BMI levels, particularly in the overweight^33^ and obesity^46^ categories, could explain the higher RRs observed in the sensitivity analysis.

The association of BMI with PASC remained consistent across outcome measures, with larger-magnitude associations for U09.9, possibly due to its specificity compared with PASC symptoms and conditions. We used both measures to enhance comprehensiveness and precision. To address implausible BMI values in EHR data, we employed the PEDSnet Data Coordinating Center algorithm,^47^ matching the closest height within 60 days of a recorded weight. While this method improves accuracy, it may not fully eliminate all inaccuracies inherent in EHR analysis.

The associations observed in our study may be explained by several plausible biological mechanisms, although they may not be pediatric-specific. First, obesity’s association with chronic inflammation^48,49^ and susceptibility to COVID-19 may involve macrophages pyroptosis,^50^ leading to prolonged systemic inflammation implicated in the genesis of PASC.^51,52^ Second, obesity is also associated with altered microbiota,^53^ and the impact of COVID-19 on the latter may influence PASC risk.^54^ Third, obesity is recognized for its propensity to dysregulate adaptive autoimmunity,^55,56^ a phenomenon documented in patients with PASC.^57,58^ This dysregulation in adaptive immune responses may be implicated in the protracted nature of observed symptoms. Furthermore, clotting and endothelial abnormalities associated with obesity could explain observed pathophysiological changes in patients with PASC.^57,59,60,61^

### Limitations

Our study has several limitations. The high obesity prevalence may indicate sample skewness, requiring caution in generalizing to the entire US pediatric population. The absence of information on modifiable risk factors like diet and physical activity, which may reduce PASC risk,^19^ limits our findings, warranting further investigations in future studies.

Potential for overclassification in pediatric PASC diagnosis due to a lack of standardized criteria is also a concern and could be mitigated by using both *ICD-10-CM* codes and PASC symptoms and conditions based on experts’ suggestions as outcomes, although this may not be comprehensive. Some symptoms we examined may overlap with those common in pediatric populations with obesity or other conditions, potentially inflating the observed associations. To address potential overlap between PASC symptoms and obesity-related conditions, we focused on new, incident cases during follow-up.

Selection bias is another concern, particularly if children with higher BMI are more likely to be hospitalized, potentially leading to an overrepresentation of severe cases. This is highlighted by literature suggesting that collider bias can distort disease risk and severity assessment.^62^ By incorporating primary care and outpatient data, we attempted to capture a broader spectrum of disease severity, although children managing COVID-19 or PASC at home might still be underrepresented.

## Conclusions

Our study found a significant association of higher pre–COVID-19 BMI with increased PASC risk in pediatric patients, highlighting the need for vigilant monitoring and personized care for those with elevated BMI. This association underscores the importance of early identification and targeted interventions to prevent long-term consequences. Public health efforts should focus on raising awareness and promoting healthy lifestyle behaviors to reduce severe outcomes. Addressing obesity as a modifiable risk factor could alleviate the burden of PASC and improve pediatric health postpandemic. Further research is needed to explore specific PASC symptoms associated with BMI.

## Notes

Supplement 1. **eTable 1.** The 26 Sites That Contributed Data in the Analysis **eMethods.** Data Description **eTable 2.** Details for the Medical Conditions in the Exclusion Criteria **eFigure.** The Participants Selection Process **eAppendix.** Statistical Methods Description **eTable 3.** Characteristics According to Missingness of BMI Status Prior to COVID-19 Infection **eTable 4.** Incident Occurrences of PASC Symptoms and Conditions Within Follow-Up Period **eTable 5.** Estimated Association of BMI Status Prior to the SARS-CoV-2 Infection and Risk of PASC by Considering the Assessment Time of BMI Status (N=105326) **eTable 6.** Estimated Association of BMI Status Prior to the SARS-CoV-2 Infection and Risk of PASC by Considering the Release Time of U09.9 (N=101143) **eTable 7.** Estimated Association of BMI Status Prior to the SARS-CoV-2 Infection and Risk of PASC by Excluding Participants Who Were Included With the Diagnosis of PASC Alone Considering the Release Time of U09.9 (N=100509) **eTable 8.** Estimated Association of BMI Status Prior to the SARS-CoV-2 Infection and Risk of PASC After Excluding Participants Confirmed by Serology Test After November 2022 (N=171978) **eTable 9.** Estimated Association of BMI Status Prior to the SARS-CoV-2 Infection and Risk of PASC After Excluding Severe or Moderate Participants (N=163094) **eTable 10.** Estimated Association of BMI Status Prior to the SARS-CoV-2 Infection and Risk of PASC Adjusting for Doses of COVID-19 Vaccine Before Infection (N=172316) **eTable 11.** Estimated Association of BMI Status Prior to the SARS-CoV-2 Infection and Risk of PASC Adjusting for Interval Since Last COVID-19 Vaccination Date (N=172316) **eTable 12.** Estimated Association of BMI Status Prior to the SARS-CoV-2 Infection and Risk of PASC Adjusting for Doses of COVID-19 Vaccine Before Infection and Interval Since Last COVID-19 Vaccination Date (N=172316) **eTable 13**. Estimated Association of BMI Status Prior to the SARS-CoV-2 Infection and Risk of PASC Adjusting Type of Insurance1 (N=140036) **eTable 14.** Estimated Association of BMI Status Prior to the SARS-CoV-2 Infection and Risk of PASC Accounting for the Clustering by Sites (N=172136) **eTable 15.** Estimated Association of BMI Status Prior to the SARS-CoV-2 Infection and Risk of PASC After Excluding Participants With Diabetes (N=169495) **eTable 16.** Estimated Association of BMI Status Prior to the SARS-CoV-2 Infection and Risk of PASC After Excluding Obese Participants Taking Weight Loss Drugs (N=169255) **eTable 17.** Estimated Association of BMI Status Prior to the SARS-CoV-2 Infection and Risk of PASC Based on Primary Care Sites (N=42470) **eTable 18.** Estimated Association of BMI Status Prior to the SARS-CoV-2 Infection and Risk of Foreign Body in Ear as a Negative Control Outcome (N=172316) **eTable 19.** Negative Control Outcomes
eReferences.

Supplement 2.
Data Sharing Statement
