# Supplementary material for: Body Mass Index and Postacute Sequelae of SARS-CoV-2 Infection in Children and Young Adults
Source: JAMA Netw Open. 2024 Oct 28;7(10):e2441970. doi: 10.1001/jamanetworkopen.2024.41970 (PMC11581483; doi:10.1001/jamanetworkopen.2024.41970)
Supplement: Supplement 1. — eTable 1. The 26 Sites That Contributed Data in the Analysis eMethods. Data Description eTable 2. Details for the Medical Conditions in the Exclusion Criteria eFigure. The Participants Selection Process eAppendix. Statistical Methods Description eTable 3. Characteristics According to Missingness of BMI Status Prior to COVID-19 Infection eTable 4. Incident Occurrences of PASC Symptoms and Conditions Within Follow-Up Period eTable 5. Estimated Association of BMI Status Prior to the SARS-CoV-2 Infection and Risk of PASC by Considering the Assessment Time of BMI Status (N=105326) eTable 6. Estimated Association of BMI Status Prior to the SARS-CoV-2 Infection and Risk of PASC by Considering the Release Time of U09.9 (N=101143) eTable 7. Estimated Association of BMI Status Prior to the SARS-CoV-2 Infection and Risk of PASC by Excluding Participants Who Were Included With the Diagnosis of PASC Alone Considering the Release Time of U09.9 (N=100509) eTable 8. Estimated Association of BMI Status Prior to the SARS-CoV-2 Infection and Risk of PASC After Excluding Participants Confirmed by Serology Test After November 2022 (N=171978) eTable 9. Estimated Association of BMI Status Prior to the SARS-CoV-2 Infection and Risk of PASC After Excluding Severe or Moderate Participants (N=163094) eTable 10. Estimated Association of BMI Status Prior to the SARS-CoV-2 Infection and Risk of PASC Adjusting for Doses of COVID-19 Vaccine Before Infection (N=172316) eTable 11. Estimated Association of BMI Status Prior to the SARS-CoV-2 Infection and Risk of PASC Adjusting for Interval Since Last COVID-19 Vaccination Date (N=172316) eTable 12. Estimated Association of BMI Status Prior to the SARS-CoV-2 Infection and Risk of PASC Adjusting for Doses of COVID-19 Vaccine Before Infection and Interval Since Last COVID-19 Vaccination Date (N=172316) eTable 13. Estimated Association of BMI Status Prior to the SARS-CoV-2 Infection and Risk of PASC Adjusting Type of Insurance1 (N=140036) eTable 14. Estim [file jamanetwopen-e2441970-s001.pdf]

Supplemental Online Content

Zhou T, Zhang B, Zhang D, et al. Body mass index and postacute sequelae of SARS-CoV-2 infection in children and young adults. *JAMA Netw. Open*. 2024;7(10):e2441970. doi:10.1001/jamanetworkopen.2024.41970

eTable 1. The 26 Sites That Contributed Data in the Analysis
eMethods. Data Description
eTable 2. Details for the Medical Conditions in the Exclusion Criteria
eFigure. The Participants Selection Process
eAppendix. Statistical Methods Description
eTable 3. Characteristics According to Missingness of BMI Status Prior to COVID-19 Infection
eTable 4. Incident Occurrences of PASC Symptoms and Conditions Within Follow-Up Period
eTable 5. Estimated Association of BMI Status Prior to the SARS-CoV-2 Infection and Risk of PASC by Considering the Assessment Time of BMI Status (N=105326)
eTable 6. Estimated Association of BMI Status Prior to the SARS-CoV-2 Infection and Risk of PASC by Considering the Release Time of U09.9 (N=101143)
eTable 7. Estimated Association of BMI Status Prior to the SARS-CoV-2 Infection and Risk of PASC by Excluding Participants Who Were Included With the Diagnosis of PASC Alone Considering the Release Time of U09.9 (N=100509)
eTable 8. Estimated Association of BMI Status Prior to the SARS-CoV-2 Infection and Risk of PASC After Excluding Participants Confirmed by Serology Test After November 2022 (N=171978)
eTable 9. Estimated Association of BMI Status Prior to the SARS-CoV-2 Infection and Risk of PASC After Excluding Severe or Moderate Participants (N=163094)
eTable 10. Estimated Association of BMI Status Prior to the SARS-CoV-2 Infection and Risk of PASC Adjusting for Doses of COVID-19 Vaccine Before Infection (N=172316)
eTable 11. Estimated Association of BMI Status Prior to the SARS-CoV-2 Infection and Risk of PASC Adjusting for Interval Since Last COVID-19 Vaccination Date (N=172316)
eTable 12. Estimated Association of BMI Status Prior to the SARS-CoV-2 Infection and Risk of PASC Adjusting for Doses of COVID-19 Vaccine Before Infection and Interval Since Last COVID-19 Vaccination Date (N=172316)
eTable 13. Estimated Association of BMI Status Prior to the SARS-CoV-2 Infection and Risk of PASC Adjusting Type of Insurance1 (N=140036)
eTable 14. Estimated Association of BMI Status Prior to the SARS-CoV-2 Infection and Risk of PASC Accounting for the Clustering by Sites (N=172136)
eTable 15. Estimated Association of BMI Status Prior to the SARS-CoV-2 Infection and Risk of PASC After Excluding Participants With Diabetes (N=169495)

© 2024 Zhou T et al. *JAMA Network Open.*

eTable 16. Estimated Association of BMI Status Prior to the SARS-CoV-2 Infection and Risk of PASC After Excluding Obese Participants Taking Weight Loss Drugs (N=169255)
eTable 17. Estimated Association of BMI Status Prior to the SARS-CoV-2 Infection and Risk of PASC Based on Primary Care Sites (N=42470)
eTable 18. Estimated Association of BMI Status Prior to the SARS-CoV-2 Infection and Risk of Foreign Body in Ear as a Negative Control Outcome (N=172316)
eTable 19. Negative Control Outcomes
eReferences.

This supplemental material has been provided by the authors to give readers additional information about their work.

© 2024 Zhou T et al. *JAMA Network Open.*

eTable 1. The 26 Sites That Contributed Data in the Analysis

ID	Site Name	City, State
1	Albert Einstein College of Medicine	Bronx, New York
2	Ann & Robert H. Lurie Children’s Hospital of Chicago	Chicago, Illinois
3	Cincinnati Children’s Hospital Medical Center	Cincinnati, Ohio
4	Children’s Hospital of Philadelphia	Philadelphia, Pennsylvania
5	Children’s National Hospital	Washington, District of Columbia
6	Colorado Children’s Hospital	Aurora, Colorado
7	Duke University Health System	Durham, North Carolina
8	Medical College of Wisconsin	Milwaukee, Wisconsin
9	Medical University of South Carolina	Charleston, South Carolina
10	Nationwide Children’s Hospital	Columbus, Ohio
11	Nemours Children’s Hospital	Orlando, Florida
12	New York University Langone Health	New York, New York
13	Northwestern University	Evanston, Illinois
14	OCHIN, Inc	Portland, Oregon
15	Seattle Children’s Hospital	Seattle, Washington
16	Stanford Children’s Health	Stanford, California
17	The Ohio State University	Columbus,Ohio
18	University of California, San Francisco	San Francisco, California
19	University of Iowa Healthcare	Iowa City, Iowa
20	University of Michigan	Ann Arbor, Michigan
21	University of Missouri	Columbia, Missouri
22	University of Nebraska Medical Center	Omaha, NE
23	UPMC-Children's Hospital of Pittsburgh	Pittsburgh, Pennsylvania
24	Vanderbilt University Medical Center	Nashville, Tennessee
25	Wake Forest University Health Sciences	Winston-Salem, NC
26	Weill Cornell Medicine	New York, New York

© 2024 Zhou T et al. *JAMA Network Open.*

eMethods. Data Description

A. Description of electronic health records (EHR) data

The real-world data utilized in our analysis is derived from electronic health records (EHRs), covering a wide range of healthcare interaction information routinely collected and stored by hospitals. This includes clinical data such as diagnoses and treatments, laboratory and test results, and administrative data including patient demographics and billing information [1]. The hospital-based EHR data from the Researching COVID to Enhance Recovery (RECOVER) Initiative COVID-19 Database served as the basis for defining and determining exposure, outcomes, and covariates. Unlike General Practitioner (GP) data, self-reported data, or external data sources, our study used the structured, standardized EHR entries made by healthcare providers within hospital settings [2]. EHR data provides a more detailed and integrated view of a patient's health status, medical history, and healthcare interactions across various providers and settings [3].

B. RECOVER population and generalizability

The National Institutes of Health (NIH) launched the new RECOVER initiative in 2021 to leverage electronic health record (EHR) data to better identify and characterize patients with post-acute sequelae of SARS-CoV-2 infection (PASC). RECOVER obtains EHRs from three large national healthcare networks within the United States, covering regional catchment areas across 41 states [4]. These networks collectively hold the EHRs of over 60 million patients, including records from more than 7 million individuals who have been affected by COVID-19 [5]. RECOVER collaborates with the National Institutes of Health's (NIH) All of Us Research Program, which contributes additional health records to this vast database [6]. Together, these sources comprise one of the world's largest collections of EHRs.

In our study, participating institutions included: Albert Einstein College of Medicine, Ann & Robert H. Lurie Children's Hospital of Chicago, Cincinnati Children's Hospital Medical Center, Children's Hospital of Philadelphia, Children's National Hospital, Colorado Children's Hospital, Duke University Health System, Medical College of Wisconsin, Medical University of South Carolina, Nationwide Children's Hospital, Nemours Children's Health System, New York University Langone Health, Northwestern University, OCHIN, Inc., Seattle Children's Hospital, Stanford Children's Health, The Ohio State University, University of California, San Francisco, University of Iowa Healthcare, University of Michigan, University of Missouri, University of Nebraska Medical Center, UPMC-Children's Hospital of Pittsburgh, Vanderbilt University Medical Center, Wake Forest University Health Sciences, Weill Cornell Medicine. For this study, we used the s9 version of the data, collected till May 2023.

C. Cohort definition and observation windows

We identified the study cohort by selecting:

- Children and adolescents aged under 21 years at the start of the study period, aligning with the American Academy of Pediatrics' definition of the pediatric population [7].
- Participants with no genetic syndromes associated with obesity or any conditions signaling a need for weight gain or a medical cause of altered weight tendencies during the baseline period.
- Participants aged above 5 at the time of assessing BMI due to their potential for more dramatic BMI variations during the baseline period.
- Users of the healthcare systems, defined as having at least one primary care visit during both baseline and follow-up periods (either in-person, via phone, or through telehealth).

To guarantee a comprehensive follow-up for all participants, we mandated that entry into the cohort—applicable to both the intervention and control groups—be timed no later than 179 days prior to the conclusion of the study period. This stipulation was critical to secure a full 179-day follow-up duration for each participant, thereby upholding the integrity of the follow-up data.

© 2024 Zhou T et al. *JAMA Network Open.*

eTable 2. Details for the Medical Conditions in the Exclusion Criteria

Condition	Condition Name
Genetic syndromes associated with obesity	MC4R deficiency, Leptin deficiency, Leptin receptor deficiency, POMC deficiency, Proprotein subtilisin or kexin type 1 deficiency, SRC1 deficiency, Prader-Willi syndrome, Alstrom syndrome, Bardet-Biedl syndrome, Smith-Magenis syndrome, SH2B1 deficiency, Sim1 deficiency, 16p11.2 microdeletion syndrome, Brain derived neurotrophic factor deficiency, Albright's hereditary osteodystrophy, Cohen syndrome, Beckwith-Wiedemann syndrome
Conditions signaling a need for weight gain or a medical cause of altered weight tendencies	Bariatric surgery, BMI less than 5th percentile for age and sex, cancer, Crohn's disease, Cushing syndrome, cystic fibrosis, eating disorder, growth hormone deficiency, HIV/AIDS, panhypopituitarism, pregnancy, sickle cell disease, ulcerative colitis

© 2024 Zhou T et al. *JAMA Network Open.*

eFigure. The Participants Selection Process

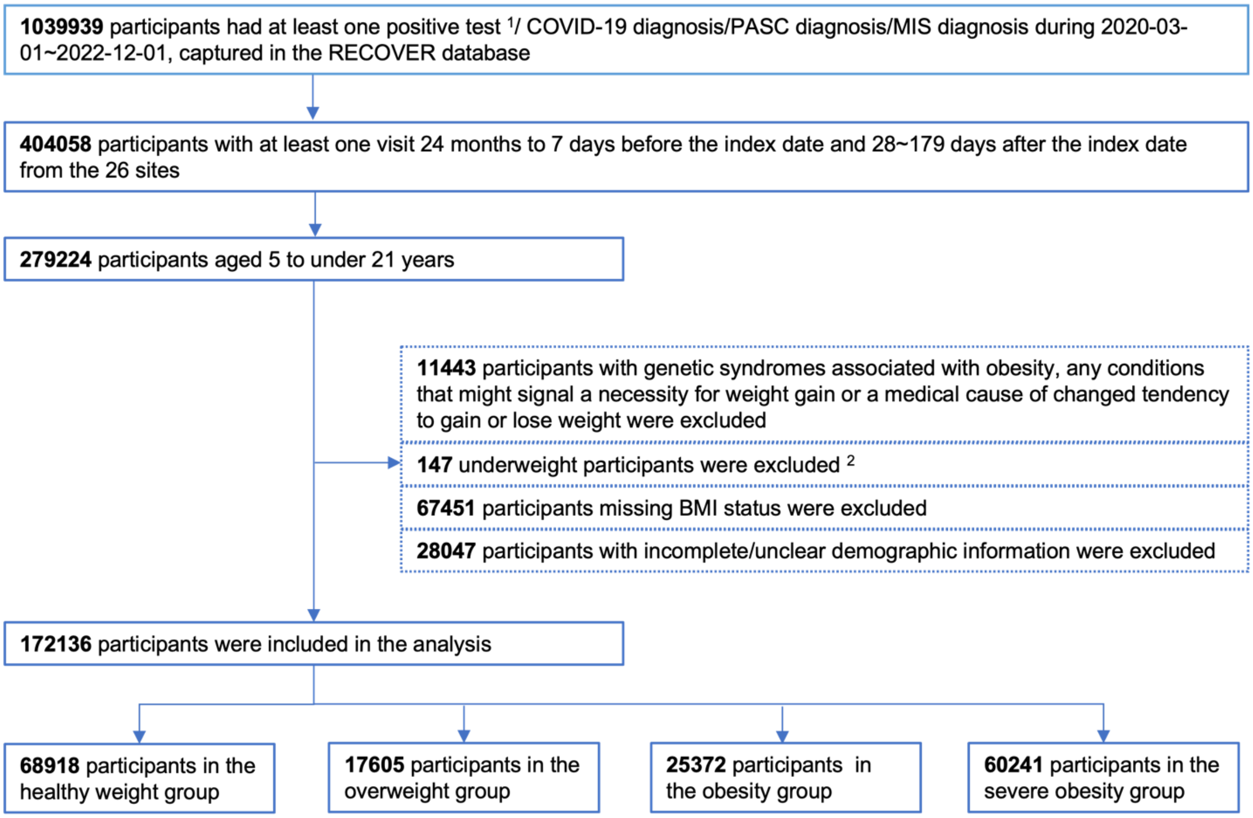

[1] Including PCR, antigen, and serology tests

[2] BMI status was assessed within 18 months before the index date, the measure closest to the index date was selected.

© 2024 Zhou T et al. *JAMA Network Open.*

eAppendix. Statistical Methods Description

A. Modified Poisson Regression

Modified Poisson Regression is a statistical method used to estimate relative risks (RR) or risk ratios for binary outcomes, especially in cohort studies or clinical trials [8]. Unlike logistic regression, which estimates odds ratios, modified Poisson regression directly estimates relative risks, which are often more interpretable in epidemiological studies where the event of interest is not rare.

Modified Poisson regression uses a sandwich estimator (also known as the Huber-White estimator) to provide robust standard errors. This adjustment accounts for the misspecification of the Poisson model for binary data and ensures correct inference [9]. It was introduced as an alternative to logistic regression when the outcome is common (incidence >10%), as odds ratios (OR) from logistic regression can overestimate the relative risk in these situations [10]. Modified Poisson regression often converges when log-binomial models (another method for estimating relative risks) fail to do so, especially with continuous covariates or multiple categorical predictors [11]. It can accommodate various types of independent variables (continuous, categorical) and can be extended to include interaction terms and random effects [12].

The model takes the form:

$$\log(\pi) = \beta_0 + \beta_1 X_1 + \beta_2 X_2 + ... + \beta_k X_k$$

Where π is the probability of the outcome, β_0 is the intercept, and β_1 to β_k are the regression coefficients for predictors X_1 to X_k.

The relative risk for a binary predictor is then estimated as:

$$RR = \exp(\beta)$$

In summary, modified Poisson regression It avoids the issues related to ORs being interpreted as RRs when the outcome is common. It is particularly useful when the sample size is large and when the probability of the event is high.

B. Trend Analysis

The trend test we used in the study is Orthogonal Trend Analysis, also known as Orthogonal Polynomial Trend Analysis or Orthogonal Polynomial Contrasts. It is a statistical method used in the analysis of variance (ANOVA) to analyze trends in data across ordered levels of a factor. This method is particularly useful in experimental designs where treatments have a natural order, such as time series, dose-response studies, or developmental stages. [13]

This approach decomposes the total variation in the data into independent (orthogonal) components, typically representing linear, quadratic, cubic, and higher-order trends. Each component is orthogonal, meaning that the components are uncorrelated and do not overlap, allowing for clear interpretation of the trends [14]. By focusing on specific trend components, this method can be more powerful in detecting patterns than omnibus F-tests, especially when the underlying trend matches one of the orthogonal components. Each trend component has a clear interpretation in terms of the shape of the relationship between the factor levels and the response variable. It can be applied to factors with equal or unequal intervals between levels, though the coefficients for the trend components differ in these cases.

Implementation:

- Define Orthogonal Polynomials: For each trend component (linear, quadratic, etc.), a set of coefficients is defined that creates an orthogonal contrast across the factor levels.
- Calculate Contrast Sums of Squares: Using these coefficients, contrast sums of squares are calculated for each trend component.

© 2024 Zhou T et al. *JAMA Network Open.*

- Test Significance: The significance of each trend component is tested using F-tests or t-tests, depending on the experimental design.
- Interpret Results: Significant trend components indicate the presence of specific patterns in the data (e.g., a significant linear trend suggests a straight-line relationship).

In summary, this technique is particularly useful in experimental and observational studies where the objective is to understand how a response variable changes systematically across levels of an independent variable. Specifically, orthogonal trend analysis provides a clear interpretation of different types of trends (e.g., linear vs. quadratic) without the confounding effects of correlations between trend components. By using orthogonal contrasts, this method allows the extraction of maximum information from the data with minimal redundancy.

This technique is particularly useful in experimental and observational studies where the objective is to understand how a response variable changes systematically across levels of an independent variable. For example, in dose-response studies, orthogonal trend analysis helps in determining whether the response follows a linear, quadratic, or higher-order trend with increasing doses. In our study, orthogonal trend analysis helps to determining whether the response follows a linear dose-response trend with increasing BMI status (i.e., healthy weight, overweight, obesity, and severe obesity) in terms of the PASC outcomes.

C. Negative control experiments

To evaluate the robustness of our pipeline, we selected from a list of 40 negative control outcomes to conduct the negative control experiments, but only one, i.e., foreign body in ear was employed. In this investigation, negative control outcomes are defined as clinical outcomes believed to have no causal relationship to the exposure. The full list of outcomes for our research was designated by pediatric physicians. A comprehensive list of these outcomes can be found in eTable 19.

For these negative control outcomes, the null hypothesis is that the exposure (i.e., elevated BMI) has no effect on these outcomes. Given the potential existence of systematic error, these negative control outcomes are used to assess the performance of our study design and contribute to calibrating our estimated effect[15, 16]. Specifically, we initially estimated the empirical null distribution of these negative control outcomes, employing the identical modified Poisson regression method.

© 2024 Zhou T et al. *JAMA Network Open.*

eTable 3. Characteristics According to Missingness of BMI Status Prior to COVID-19 Infection[a]

Characteristics	Missing BMI status, No (%)		
	Yes (N=67451)	No (N=172136)	*P* value [b]
Mean age (SD), yr [c]	10.07 (6.41)	13.06 (4.37)	<0.001
Sex			
Male	33772 (50.07)	81949 (47.61)	<0.001
Female	33679 (49.93)	90187 (52.39)	<0.001
Race			
Hispanic	15476 (22.94)	42982 (24.97)	<0.001
Non-Hispanic Black	13475 (19.98)	33065 (19.21)	<0.001
Non-Hispanic White	35156 (52.12)	87275 (50.70)	<0.001
Other [d]	3344 (4.96)	8814 (5.12)	0.105
Predominant variant			
Pre-Alpha	17496 (25.94)	45440 (26.40)	0.022
Alpha	3795 (5.63)	8555 (4.97)	<0.001
Delta	17527 (25.98)	42490 (24.68)	<0.001
Omicron	28633 (42.45)	75651 (43.95)	<0.001
PMCA			
None	58732 (87.07)	105294 (61.17)	<0.001
Noncomplex	6304 (9.35)	40135 (23.32)	<0.001
Complex	2415 (3.58)	26707 (15.52)	<0.001
Severity			
Asymptomatic	40799 (60.49)	102366 (59.47)	<0.001
Mild	21787 (32.30)	60728 (35.28)	<0.001
Moderate	3305 (4.90)	5704 (3.31)	<0.001
Severe	1560 (2.31)	3338 (1.94)	<0.001
Numbers of negative COVID-19 tests			
0	43079 (63.87)	104312 (60.60)	<0.001
1	14719 (21.82)	36964 (21.47)	0.063
≥2	9653 (14.31)	30860 (17.93)	<0.001
Numbers of ED visits			
0	46010 (68.21)	129862 (75.44)	<0.001
1	12164 (18.03)	22896 (13.30)	<0.001
2	4670 (6.92)	9188 (5.30)	<0.001
≥3	4670 (6.83)	10260 (5.96)	<0.001
Numbers of IPD visits			
0	65195 (96.66)	158826 (92.27)	<0.001
1	1912 (2.83)	8404 (4.88)	<0.001
2	214 (0.32)	2383 (1.38)	<0.001
≥3	130 (0.19)	2523 (1.47)	<0.001
Numbers of OPD visits			
0	28339 (42.01)	8830 (5.13)	<0.001
1	14479 (21.47)	15274 (8.87)	<0.001
2	7976 (11.82)	19255 (11.19)	<0.001
≥3	16657 (24.69)	128777 (74.81)	<0.001
Numbers of medications or prescriptions			
0	31523 (46.73)	28421 (16.51)	<0.001
1	9749 (14.45)	19251 (11.18)	<0.001
2	6827 (10.12)	17632 (10.24)	0.381
≥3	19352 (28.69)	106832 (62.06)	<0.001
PASC (U09.9)	532 (0.79)	1402 (0.81)	0.543
Any occurrence of PASC symptoms	24232 (35.93)	74317 (43.17)	<0.001
Median total occurrence of PASC symptoms (IQR)	0 (0-14)	0 (0-15)	<0.001

© 2024 Zhou T et al. *JAMA Network Open.*

Abbreviations: BMI, body mass index; ED, Emergency department; IPD, Inpatient department; IQR, interquartile range; OPD, Outpatient department; PASC, post-acute sequelae of SARS-CoV-2 infection; PMCA, Pediatric Medical Complexity; SD, Standard deviation.

[a] Percentages may not total 100 because of rounding.

[b] Student's t test for age and total occurrence of PASC symptoms, contingency chi-square test for the other categorical variables.

[c] Referred to the cohort entry age.

[c] Other race/ethnicity included Asian American/Pacific Islander.

© 2024 Zhou T et al. *JAMA Network Open.*

eTable 4. Incident Occurrences of PASC Symptoms and Conditions Within Follow-Up Period[a]

PASC symptoms and conditions	Incident count	Total count	Incidence (%)
**Any occurrences of symptoms and conditions [b]**	**16636**	**63046**	**26.387**
Abdominal pain	6370	152662	4.173
Abnormal liver enzymes	531	170595	0.311
Acute kidney injury	367	171175	0.214
Acute respiratory distress syndrome	39	172098	0.023
Arrythmias	2620	164984	1.588
Cardiovascular signs and symptoms	1750	166377	1.052
Changes in taste and smell	427	171446	0.249
Chest pain	3402	163905	2.076
Cognitive function	604	169881	0.356
Fatigue and malaise	3535	162370	2.177
Fever and chills	5551	156957	3.537
Fluid and electrolyte imbalances	754	170036	0.443
Generalized pain	2930	163575	1.791
Hair loss	524	170953	0.307
headache	5465	153301	3.565
Heart disease	653	170130	0.384
Mental health disorders	9548	128024	7.458
musculoskeletal	7586	145561	5.212
myocarditis	94	172074	0.055
myositis	39	172031	0.023
POTS	2701	165174	1.635
Respiratory signs and symptoms	10967	134345	8.163
Skin symptoms	5110	154988	3.297
Thrombophlebitis and thromboembolism	192	171667	0.112

Abbreviation: PASC, post-acute sequelae of SARS-CoV-2 infection; POT, postural orthostatic tachycardia syndrome or dysautonomia.

[a] The incident occurrences were assessed within the follow-up period, but that did not occur during the baseline period.

[b] Any occurrences of symptoms and conditions was defined as any incident occurrences of the 24 PASC symptoms and conditions within the follow-up period, but that did not occur during the baseline period.

© 2024 Zhou T et al. *JAMA Network Open.*

eTable 5. Estimated Association of BMI Status Prior to the SARS-CoV-2 Infection and Risk of PASC by Considering the Assessment Time of BMI Status [ab] (N=105326)

Outcome	BMI Status	Incidental/total COVID, No (%) [c]	RR (95% CI)	*P* for trend
PASC (U09.9)	Healthy weight	338/42404 (0.8)	1 [Reference]	0.01
	Overweight	100/11049 (0.9)	1.153 (0.924, 1.439)	
	Obesity	137/15879 (0.9)	**1.305 (1.069, 1.592)**	
	Severe obesity	352/35994 (1.0)	**1.403 (1.201, 1.638)**	
PASC symptoms and conditions	Any Occurrences			<0.001
	Healthy weight	19292/42404 (45.5)	1 [Reference]	
	Overweight	5270/11049 (47.7)	1.052 (0.987, 1.121)	
	Obesity	7520/15879 (47.4)	**1.109 (1.050, 1.172)**	
	Severe obesity	17473/35994 (48.5)	**1.171 (1.121, 1.223)**	
	Total Occurrences (median)			<0.001
	Healthy weight	0	1 [Reference]	
	Overweight	0	**1.099(1.025, 1.179)**	
	Obesity	0	**1.146(1.080, 1.216)**	
	Severe obesity	0	**1.186 (1.132, 1.244)**	

Abbreviation: BMI, body mass index; CI, confidence interval; PASC, post-acute sequelae of SARS-CoV-2 infection; RR, relative risk.

[a] The BMI status was assessed within 6 months before cohort entry rather than 18 months before cohort entry.

[b] Adjusted for age assessed BMI and entered cohort (continuous), sex, race/ethnicity, PMCA index, predominant variant, acute COVID-19 severity, numbers of emergency department visits, outpatient department visits, inpatient department visits, medications or prescriptions, and negative COVID-19 tests.

[c] Incident referred to the count of participants developed the outcome we interested in, total COVID referred to the count of the participants in the corresponding group, and the value in the bracket referred to the percentage of the groups who developed the outcome we interested in.

© 2024 Zhou T et al. *JAMA Network Open.*

eTable 6. Estimated Association of BMI Status Prior to the SARS-CoV-2 Infection and Risk of PASC by Considering the Release Time of U09.9 [ab] (N=101143)

Outcome	BMI Status	Incident/total COVID, No (%) [c]	RR (95% CI)	*P* for trend
PASC (U09.9)	Healthy weight	427/40490 (1.1)	1 [Reference]	0.004
	Overweight	112/10205 (1.1)	1.056 (0.860, 1.296)	
	Obesity	158/15335 (1.0)	**1.220 (1.017, 1.464)**	
	Severe obesity	451/35113 (1.3)	**1.419 (1.236, 1.629)**	
PASC symptoms and conditions	Any Occurrences			
	Healthy weight	17518/40490 (43.3)	1 [Reference]	<0.001
	Overweight	4642/10205 (45.5)	1.026 (0.964, 1.091)	
	Obesity	7086/15335 (46.2)	**1.147 (1.091, 1.207)**	
	Severe obesity	16659/35113 (47.4)	**1.203 (1.156, 1.253)**	
	Total Occurrences (median)			
	Healthy weight	0	1 [Reference]	<0.001
	Overweight	0	1.051 (0.982, 1.124)	
	Obesity	0	**1.178 (1.115, 1.244)**	
	Severe obesity	0	**1.214 (1.162, 1.268)**	

Abbreviation: BMI, body mass index; CI, confidence interval; PASC, post-acute sequelae of SARS-CoV-2 infection; RR, relative risk.

[a] The cohort entry date was restricted to after October 1, 2021 when the U09.9 was released.

[b] Adjusted for age assessed BMI and entered cohort (continuous), sex, race/ethnicity, PMCA index, predominant variant, acute COVID-19 severity, numbers of emergency department visits, outpatient department visits, inpatient department visits, medications or prescriptions, and negative COVID-19 tests.

[c] Incident referred to the count of participants developed the outcome we interested in, total COVID referred to the count of the participants in the corresponding group, and the value in the bracket referred to the percentage of the groups who developed the outcome we interested in.

© 2024 Zhou T et al. *JAMA Network Open.*

eTable 7. Estimated Association of BMI Status Prior to the SARS-CoV-2 Infection and Risk of PASC by Excluding Participants Who Were Included With the Diagnosis of PASC Alone Considering the Release Time of U09.9 [ab] (N=100509)

Outcome	BMI Status	Incident/total COVID, No (%) [c]	RR (95% CI)	*P* for trend
PASC (U09.9)	Healthy weight	182/40313 (0.5)	1 [Reference]	0.006
	Overweight	43/10134 (0.4)	1.056 (0.861, 1.295)	
	Obesity	64/15246 (0.4)	**1.206 (1.006, 1.446)**	
	Severe obesity	156/34816 (0.4)	**1.404 (1.223, 1.611)**	
PASC symptoms and conditions	Any Occurrences			
	Healthy weight	17353/40313 (43.0)	1 [Reference]	<0.001
	Overweight	4580/10134 (45.2)	1.026 (0.964, 1.091)	
	Obesity	7010/15246 (46.0)	**1.147 (1.090, 1.207)**	
	Severe obesity	16413/34816 (47.1)	**1.203 (1.156, 1.253)**	
	Total Occurrences (median)			
	Healthy weight	0	1 [Reference]	<0.001
	Overweight	0	1.050 (0.982, 1.124)	
	Obesity	0	**1.177 (1.114, 1.243)**	
	Severe obesity	0	**1.214 (1.162, 1.268)**	

Abbreviation: BMI, body mass index; CI, confidence interval; PASC, post-acute sequelae of SARS-CoV-2 infection; RR, relative risk.
[a] Participants who were included with the diagnosis of PASC alone were excluded, where the entrance date was also constrained to after October 1, 2021 when the U09.9 was released.
[b] Adjusted for age assessed BMI and entered cohort (continuous), sex, race/ethnicity, PMCA index, predominant variant, acute COVID-19 severity, numbers of emergency department visits, outpatient department visits, inpatient department visits, medications or prescriptions, and negative COVID-19 tests.

© 2024 Zhou T et al. *JAMA Network Open.*

eTable 8. Estimated Association of BMI Status Prior to the SARS-CoV-2 Infection and Risk of PASC After Excluding Participants Confirmed by Serology Test After November 2022 (N=171978)[a]

Outcome	BMI Status	Incident/total COVID, No (%) [b]	RR (95% CI)	*P* for trend
PASC (U09.9)	Healthy weight	506/68827 (0.7)	1 [Reference]	0.001
	Overweight	137/17585 (0.8)	1.064 (0.882, 1.284)	
	Obesity	196/25333 (0.8)	**1.259 (1.067, 1.485)**	
	Severe obesity	551/60233 (0.9)	**1.437 (1.267, 1.630)**	
PASC symptoms and conditions	Any Occurrences			
	Healthy weight	28600/68827 (41.6)	1 [Reference]	<0.001
	Overweight	7620/17585 (43.3)	1.028 (0.981, 1.079)	
	Obesity	11050/25333 (43.6)	**1.108 (1.064, 1.154)**	
	Severe obesity	26919/60233 (44.7)	**1.177 (1.140, 1.215)**	
	Total Occurrences (median)			
	Healthy weight	0	1 [Reference]	<0.001
	Overweight	0	1.049 (0.996, 1.104)	
	Obesity	0	**1.137 (1.088, 1.188)**	
	Severe obesity	0	**1.186 (1.146, 1.228)**	

Abbreviation: BMI, body mass index; CI, confidence interval; PASC, post-acute sequelae of SARS-CoV-2 infection; RR, relative risk.

[a] Adjusted for age assessed BMI and entered cohort (continuous), sex, race/ethnicity, PMCA index, predominant variant, acute COVID-19 severity, numbers of emergency department visits, outpatient department visits, inpatient department visits, medications or prescriptions, and negative COVID-19 tests.

[b] Incident referred to the count of participants developed the outcome we interested in, total COVID referred to the count of the participants in the corresponding group, and the value in the bracket referred to the percentage of the groups who developed the outcome we interested in.

© 2024 Zhou T et al. *JAMA Network Open.*

eTable 9. Estimated Association of BMI Status Prior to the SARS-CoV-2 Infection and Risk of PASC After Excluding Severe or Moderate Participants (N=163094)[a]

Outcome	BMI Status	Incident/total COVID, No (%) [b]	RR (95% CI)	*P* for trend
PASC (U09.9)	Healthy weight	476/64674 (0.7)	1 [Reference]	0.001
	Overweight	131/16482 (0.8)	1.085 (0.895, 1.315)	
	Obesity	186/23837 (0.8)	**1.273 (1.074, 1.508)**	
	Severe obesity	526/58101 (0.9)	**1.440 (1.264, 1.640)**	
PASC symptoms and conditions	Any Occurrences			
	Healthy weight	26269/64674 (40.6)	1 [Reference]	<0.001
	Overweight	6991/16482 (42.4)	1.027 (0.978, 1.079)	
	Obesity	10246/23837 (43.0)	**1.106 (1.061, 1.153)**	
	Severe obesity	25631/58101 (44.1)	**1.175 (1.137, 1.214)**	
	Total Occurrences (median)			
	Healthy weight	0	1 [Reference]	<0.001
	Overweight	0	1.044 (0.990, 1.101)	
	Obesity	0	**1.137 (1.087, 1.190)**	
	Severe obesity	0	**1.184 (1.143, 1.226)**	

Abbreviation: BMI, body mass index; CI, confidence interval; PASC, post-acute sequelae of SARS-CoV-2 infection; RR, relative risk.

[a] Adjusted for age assessed BMI and entered cohort (continuous), sex, race/ethnicity, PMCA index, predominant variant, numbers of emergency department visits, outpatient department visits, inpatient department visits, medications or prescriptions, and negative COVID-19 tests.

[b] Incident referred to the count of participants developed the outcome we interested in, total COVID referred to the count of the participants in the corresponding group, and the value in the bracket referred to the percentage of the groups who developed the outcome we interested in.

© 2024 Zhou T et al. *JAMA Network Open.*

eTable 10. Estimated Association of BMI Status Prior to the SARS-CoV-2 Infection and Risk of PASC Adjusting for Doses of COVID-19 Vaccine Before Infection (N=172316)[a]

Outcome	BMI Status	Incident/total COVID, No (%) [b]	RR (95% CI)	*P* for trend
PASC (U09.9)	Healthy weight	514/68918 (0.7)	1 [Reference]	0.001
	Overweight	137/17605 (0.8)	1.046 (0.868, 1.262)	
	Obesity	199/25372 (0.8)	**1.251 (1.062, 1.475)**	
	Severe obesity	552/60241 (0.9)	**1.422 (1.254, 1.611)**	
PASC symptoms and conditions	Any Occurrences			
	Healthy weight	28674/68918 (41.6)	1 [Reference]	<0.001
	Overweight	7637/17605 (43.4)	1.029 (0.982, 1.079)	
	Obesity	11081/25372 (43.7)	**1.106 (1.062, 1.152)**	
	Severe obesity	26925/60241 (44.7)	**1.175 (1.138, 1.213)**	
	Total Occurrences (median)			
	Healthy weight	0	1 [Reference]	<0.001
	Overweight	0	1.052 (0.999, 1.108)	
	Obesity	0	**1.134 (1.086, 1.186)**	
	Severe obesity	0	**1.182 (1.142, 1.223)**	

Abbreviation: BMI, body mass index; CI, confidence interval; PASC, post-acute sequelae of SARS-CoV-2 infection; RR, relative risk.

[a] Adjusted for age assessed BMI and entered cohort (continuous), sex, race/ethnicity, PMCA index, predominant variant, acute COVID-19 severity, numbers of emergency department visits, outpatient department visits, inpatient department visits, medications or prescriptions, negative COVID-19 tests, and doses of COVID-19 vaccine before infection.

[b] Incident referred to the count of participants developed the outcome we interested in, total COVID referred to the count of the participants in the corresponding group, and the value in the bracket referred to the percentage of the groups who developed the outcome we interested in.

© 2024 Zhou T et al. *JAMA Network Open.*

eTable 11. Estimated Association of BMI Status Prior to the SARS-CoV-2 Infection and Risk of PASC Adjusting for Interval Since Last COVID-19 Vaccination Date (N=172316)[a]

Outcome	BMI Status	Incident/total COVID, No (%) [b]	RR (95% CI)	*P* for trend
PASC (U09.9)	Healthy weight	514/68918 (0.7)	1 [Reference]	0.001
	Overweight	137/17605 (0.8)	1.046 (0.867, 1.261)	
	Obesity	199/25372 (0.8)	**1.249 (1.060, 1.472)**	
	Severe obesity	552/60241 (0.9)	**1.420 (1.253, 1.610)**	
PASC symptoms and conditions	Any Occurrences			
	Healthy weight	28674/68918 (41.6)	1 [Reference]	<0.001
	Overweight	7637/17605 (43.4)	1.029 (0.981, 1.079)	
	Obesity	11081/25372 (43.7)	**1.106 (1.062, 1.152)**	
	Severe obesity	26925/60241 (44.7)	**1.175 (1.138, 1.213)**	
	Total Occurrences (median)			
	Healthy weight	0	1 [Reference]	<0.001
	Overweight	0	1.052 (0.999, 1.108)	
	Obesity	0	**1.135 (1.086, 1.186)**	
	Severe obesity	0	**1.182 (1.142, 1.223)**	

Abbreviation: BMI, body mass index; CI, confidence interval; PASC, post-acute sequelae of SARS-CoV-2 infection; RR, relative risk.

[a] Adjusted for age assessed BMI and entered cohort (continuous), sex, race/ethnicity, PMCA index, predominant variant, acute COVID-19 severity, numbers of emergency department visits, outpatient department visits, inpatient department visits, medications or prescriptions, negative COVID-19 tests, interval since last COVID-19 vaccination date.

[b] Incident referred to the count of participants developed the outcome we interested in, total COVID referred to the count of the participants in the corresponding group, and the value in the bracket referred to the percentage of the groups who developed the outcome we interested in.

© 2024 Zhou T et al. *JAMA Network Open.*

eTable 12. Estimated Association of BMI Status Prior to the SARS-CoV-2 Infection and Risk of PASC Adjusting for Doses of COVID-19 Vaccine Before Infection and Interval Since Last COVID-19 Vaccination Date (N=172316)[a]

Outcome	BMI Status	Incident/total COVID, No (%) [b]	RR (95% CI)	*P* for trend
PASC (U09.9)	Healthy weight	514/68918 (0.7)	1 [Reference]	0.001
	Overweight	137/17605 (0.8)	1.046 (0.867, 1.262)	
	Obesity	199/25372 (0.8)	**1.251 (1.062, 1.475)**	
	Severe obesity	552/60241 (0.9)	**1.422 (1.254, 1.611)**	
PASC symptoms and conditions	Any Occurrences			
	Healthy weight	28674/68918 (41.6)	1 [Reference]	<0.001
	Overweight	7637/17605 (43.4)	1.029 (0.982, 1.079)	
	Obesity	11081/25372 (43.7)	**1.106 (1.062, 1.152)**	
	Severe obesity	26925/60241 (44.7)	**1.175 (1.138, 1.213)**	
	Total Occurrences (median)			
	Healthy weight	0	1 [Reference]	<0.001
	Overweight	0	1.052 (0.999, 1.108)	
	Obesity	0	**1.135 (1.086, 1.186)**	
	Severe obesity	0	**1.182 (1.142, 1.223)**	

Abbreviation: BMI, body mass index; CI, confidence interval; PASC, post-acute sequelae of SARS-CoV-2 infection; RR, relative risk.

[a] Adjusted for age assessed BMI and entered cohort (continuous), sex, race/ethnicity, PMCA index, predominant variant, acute COVID-19 severity, numbers of emergency department visits, outpatient department visits, inpatient department visits, medications or prescriptions, negative COVID-19 tests, doses of COVID-19 vaccine before infection and interval since last COVID-19 vaccination date.

[b] Incident referred to the count of participants developed the outcome we interested in, total COVID referred to the count of the participants in the corresponding group, and the value in the bracket referred to the percentage of the groups who developed the outcome we interested in.

© 2024 Zhou T et al. *JAMA Network Open.*

eTable 13. Estimated Association of BMI Status Prior to the SARS-CoV-2 Infection and Risk of PASC Adjusting Type of Insurance (N=140036)[a]

Outcome	BMI Status	Incident/total COVID, No (%) [b]	RR (95% CI)	*P* for trend
PASC (U09.9)	Healthy weight	396/54114 (0.7)	1 [Reference]	0.002
	Overweight	117/14233 (0.8)	1.161 (0.946, 1.426)	
	Obesity	166/21047 (0.8)	**1.372 (1.142, 1.649)**	
	Severe obesity	460/50642 (0.9)	**1.575 (1.339, 1.851)**	
PASC symptoms and conditions	Any Occurrences			
	Healthy weight	22624/54114 (41.8)	1 [Reference]	<0.001
	Overweight	6197/14233 (43.5)	1.018 (0.965,1.075)	
	Obesity	9037/21047 (42.9)	**1.056 (1.008,1.106)**	
	Severe obesity	22482/50642 (44.4)	**1.064 (1.021,1.109)**	
	Total Occurrences (median)			
	Healthy weight	0	1 [Reference]	<0.001
	Overweight	0	1.031 (0.972,1.094)	
	Obesity	0	**1.072 (1.019,1.127)**	
	Severe obesity	0	**1.066 (1.019,1.115)**	

Abbreviation: BMI, body mass index; CI, confidence interval; PASC, post-acute sequelae of SARS-CoV-2 infection; RR, relative risk.

[a] Adjusted for age assessed BMI and entered cohort (continuous), sex, race/ethnicity, PMCA index, predominant variant, acute COVID-19 severity, numbers of emergency department visits, outpatient department visits, inpatient department visits, medications or prescriptions, negative COVID-19 tests, and type of insurance.

[b] Incident referred to the count of participants developed the outcome we interested in, total COVID referred to the count of the participants in the corresponding group, and the value in the bracket referred to the percentage of the groups who developed the outcome we interested in.

© 2024 Zhou T et al. *JAMA Network Open.*

eTable 14. Estimated Association of BMI Status Prior to the SARS-CoV-2 Infection and Risk of PASC Account for the Clustering by Sites (N=172136)[a]

Outcome	BMI Status	Incident/total COVID, No (%) [b]	RR (95% CI)	*P* for trend
PASC (U09.9)	Healthy weight	514/68918 (0.7)	1 [Reference]	<0.001
	Overweight	137/17605 (0.8)	1.047 (0.868, 1.262)	
	Obesity	199/25372 (0.8)	1.136 (0.960, 1.345)	
	Severe obesity	552/60241 (0.9)	1.101 (0.925, 1.312)	
PASC symptoms and conditions	Any Occurrences			
	Healthy weight	28674/68918 (41.6)	1 [Reference]	<0.001
	Overweight	7637/17605 (43.4)	1.022 (0.975, 1.071)	
	Obesity	11081/25372 (43.7)	**1.057 (1.014, 1.103)**	
	Severe obesity	26925/60241 (44.7)	**1.071 (1.022, 1.123)**	
	Total Occurrences (median)			
	Healthy weight	0	1 [Reference]	<0.001
	Overweight	0	**1.041 (0.989, 1.096)**	
	Obesity	0	**1.084 (1.036, 1.135)**	
	Severe obesity	0	**1.082 (1.028, 1.138)**	

Abbreviation: BMI, body mass index; CI, confidence interval; PASC, post-acute sequelae of SARS-CoV-2 infection; RR, relative risk.

[a] Adjusted for age assessed BMI and entered cohort (continuous), sex, race/ethnicity, PMCA index, predominant variant, acute COVID-19 severity, numbers of emergency department visits, outpatient department, inpatient department, medications or prescriptions, negative COVID-19 tests, and site index.

[b] Incident referred to the count of participants developed the outcome we interested in, total COVID referred to the count of the participants in the corresponding group, and the value in the bracket referred to the percentage of the groups who developed the outcome we interested in.

© 2024 Zhou T et al. *JAMA Network Open.*

eTable 15. Estimated Association of BMI Status Prior to the SARS-CoV-2 Infection and Risk of PASC After Excluding Participants With Diabetes (N=169495)[a]

Outcome	BMI Status	Incident/total COVID, No (%) [b]	RR (95% CI)	*P* for trend
PASC (U09.9)	Healthy weight	508/67978 (0.7)	1 [Reference]	0.001
	Overweight	136/17244 (0.8)	1.058 (0.876, 1.276)	
	Obesity	196/24951 (0.8)	**1.258 (1.066, 1.484)**	
	Severe obesity	548/59322 (0.9)	**1.433 (1.263, 1.626)**	
PASC symptoms and conditions	Any Occurrences			
	Healthy weight	28302/67978 (41.6)	1 [Reference]	<0.001
	Overweight	7487/17244 (43.4)	1.032 (0.984, 1.083)	
	Obesity	11894/24951 (43.7)	**1.111 (1.067, 1.157)**	
	Severe obesity	26486/59322 (44.6)	**1.178 (1.141, 1.217)**	
	Total Occurrences (median)			
	Healthy weight	0	1 [Reference]	<0.001
	Overweight	0	1.052 (0.999, 1.108)	
	Obesity	0	**1.138 (1.089, 1.189)**	
	Severe obesity	0	**1.186 (1.146, 1.228)**	

Abbreviation: BMI, body mass index; CI, confidence interval; PASC, post-acute sequelae of SARS-CoV-2 infection; RR, relative risk.

[a] Adjusted for age assessed BMI and entered cohort (continuous), sex, race/ethnicity, PMCA index, predominant variant, acute COVID-19 severity, numbers of emergency department visits, outpatient department visits, inpatient department visits, medications or prescriptions, and negative COVID-19 tests

[b] Incident referred to the count of participants developed the outcome we interested in, total COVID referred to the count of the participants in the corresponding group, and the value in the bracket referred to the percentage of the groups who developed the outcome we interested in.

© 2024 Zhou T et al. *JAMA Network Open.*

eTable 16. Estimated Association of BMI Status Prior to the SARS-CoV-2 Infection and Risk of PASC After Excluding Obese Participants Taking Weight-Loss Drugs (N=169255)[ab]

Outcome	BMI Status	Incident/total COVID, No (%) [c]	RR (95% CI)	*P* for trend
PASC (U09.9)	Healthy weight	514/68918 (0.7)	1 [Reference]	0.002
	Overweight	137/17605 (0.8)	1.046 (0.867, 1.261)	
	Obesity	189/24432 (0.8)	**1.256 (1.062, 1.485)**	
	Severe obesity	531/58300 (0.9)	**1.429 (1.259, 1.623)**	
PASC symptoms and conditions	Any Occurrences			<0.001
	Healthy weight	28674/68918 (41.6)	1 [Reference]	
	Overweight	7637/17605 (43.4)	1.030 (0.982, 1.080)	
	Obesity	10543/24432 (43.2)	**1.115 (1.070, 1.161)**	
	Severe obesity	25800/58300 (44.3)	**1.184 (1.146, 1.222)**	
	Total Occurrences (median)			<0.001
	Healthy weight	0	1 [Reference]	
	Overweight	0	**1.053 (1.000, 1.109)**	
	Obesity	0	**1.140 (1.091, 1.192)**	
	Severe obesity	0	**1.192 (1.151, 1.234)**	

Abbreviation: BMI, body mass index; CI, confidence interval; PASC, post-acute sequelae of SARS-CoV-2 infection; RR, relative risk.

[a] Weight-loss drugs included Metformin, Orlistat, Liraglutide, Exenatide, Dulaglutide, Semaglutide, Setmelanotide, Phentermine Topiramate.

[b] Adjusted for age assessed BMI and entered cohort (continuous), sex, race/ethnicity, PMCA index, predominant variant, acute COVID-19 severity, numbers of emergency department visits, outpatient department visits, inpatient department visits, medications or prescriptions, and negative COVID-19 tests.

[c] Incident referred to the count of participants developed the outcome we interested in, total COVID referred to the count of the participants in the corresponding group, and the value in the bracket referred to the percentage of the groups who developed the outcome we interested in.

© 2024 Zhou T et al. *JAMA Network Open.*

eTable 17. Estimated Association of BMI Status Prior to the SARS-CoV-2 Infection and Risk of PASC Based on Primary Care Sites (N=42470) [a]

Outcome	BMI Status	Incident/total COVID, No (%) [b]	RR (95% CI)	*P* for trend
PASC (U09.9)	Healthy weight	172/25890 (0.7)	1 [Reference]	0.043
	Overweight	54/7083 (0.8)	1.175 (0.869, 1.590)	
	Obesity	63/7674 (0.8)	**1.450 (1.082, 1.944)**	
	Severe obesity	18/1823 (1.0)	1.137 (0.699, 1.848)	
PASC symptoms and conditions	Any Occurrences			
	Healthy weight	9335/25890 (36.1)	1 [Reference]	<0.001
	Overweight	2684/7083 (37.9)	1.057 (0.978, 1.141)	
	Obesity	3010/7674 (39.2)	**1.080 (1.002, 1.164)**	
	Severe obesity	817/1823 (44.8)	**1.106 (0.942, 1.299)**	
	Total Occurrences (median)			
	Healthy weight	0	1 [Reference]	<0.001
	Overweight	0	**1.102 (1.013, 1.198)**	
	Obesity	0	**1.118 (1.029, 1.213)**	
	Severe obesity	0	1.169 (0.981, 1.392)	

Abbreviation: BMI, body mass index; CI, confidence interval; PASC, post-acute sequelae of SARS-CoV-2 infection; RR, relative risk.

[a] Adjusted for age assessed BMI and entered cohort (continuous), sex, race/ethnicity, PMCA index, predominant variant, acute COVID-19 severity, numbers of emergency department visits, outpatient department visits, inpatient department visits, medications or prescriptions, and negative COVID-19 tests.

[b] Incident referred to the count of participants developed the outcome we interested in, total COVID referred to the count of the participants in the corresponding group, and the value in the bracket referred to the percentage of the groups who developed the outcome we interested in.

© 2024 Zhou T et al. *JAMA Network Open.*

eTable 18. Estimated Association of BMI Status Prior to the SARS-CoV-2 Infection and Risk of Foreign Body in Ear as a Negative Control Outcome (N=172316) [a]

BMI Status	Incident/total COVID (%), No (%) [b]	RR (95% CI)	*P* for trend
Healthy weight	6/68918 (0.0)	1 [Reference]	0.852
Overweight	4/17605 (0.0)	2.617 (0.705, 9.706)	
Obesity	3/25372 (0.0)	0.942 (0.245, 3.622)	
Severe obesity	5/60241 (0.0)	0.790 (0.246, 2.536)	

Abbreviation: BMI, body mass index; CI, confidence interval; RR, relative risk.

[a] Adjusted for age assessed BMI and entered cohort (continuous), sex, race/ethnicity, PMCA index, predominant variant, acute COVID-19 severity, numbers of emergency department visits, outpatient department visits, inpatient department visits, medications or prescriptions, and negative COVID-19 tests.

[b] Incident referred to the count of participants developed the outcome we interested in, total COVID referred to the count of the participants in the corresponding group, and the value in the bracket referred to the percentage of the groups who developed the outcome we interested in.

© 2024 Zhou T et al. *JAMA Network Open.*

eTable 19. Negative control outcomes

Health conditions
Wax in ear/impacted cerumen
Snoring/Obstructive sleep apnea
Contact dermatitis
Injury of head
Diaper rash
Seizure
Speech delay
Autism/Autistic disorder
Displacements - bone
Closed fractue of distal end of radius
Acne
Falls
Visual testing abnormal
Sprain of ankle
Concussion
Impetigo
Scoliosis
Foot pain
Injury of free lower limb
Injury of. Upper extremity
Speech dysfunction
Umbilical hernia
Insect bite
Myopia
Injury of finger
Injury of right leg
Astigmatism
Injury of left leg
Tinea capitis
Obesity
Injury of right hand
Tinea corporis
Epilepsy
Tongue tie
Plagiocephaly
Inguinal hernia
Pain in wrist
Closed injury of head
Foreign body in ear

© 2024 Zhou T et al. *JAMA Network Open.*

Injury of right foot

© 2024 Zhou T et al. *JAMA Network Open.*

eReferences

[1] Jensen PB, Jensen LJ, Brunak S. Mining electronic health records: towards better research applications and clinical care. Nat Rev Genet. 2012 May 2;13(6):395-405.

[2] Weiskopf NG, Weng C. Methods and dimensions of electronic health record data quality assessment: enabling reuse for clinical research. J Am Med Inform Assoc. 2013 Jan 1;20(1):144-51.

[3] Casey JA, Schwartz BS, Stewart WF, Adler NE. Using Electronic Health Records for Population Health Research: A Review of Methods and Applications. Annu Rev Public Health. 2016;37:61-81.

[4] RECOVER. Homepage. August 22, 2024. Accessed August 24, 2023. https://recovercovid.org/

[5] Pfaff ER, Girvin AT, Bennett TD, et al; N3C Consortium. Identifying who has long COVID in the USA: a machine learning approach using N3C data. Lancet Digit Health. 2022 Jul;4(7):e532-e541.

[6] All of Us Research Program Investigators; Denny JC, Rutter JL, Goldstein DB, et al. The "All of Us" Research Program. N Engl J Med. 2019 Aug 15;381(7):668-676.

[7] Hardin AP, Hackell JM; COMMITTEE ON PRACTICE AND AMBULATORY MEDICINE. Age Limit of Pediatrics. Pediatrics. 2017 Sep;140(3):e20172151.

[8] Zou G. A modified poisson regression approach to prospective studies with binary data. Am J Epidemiol. 2004 Apr 1;159(7):702-6.

[9] Zou GY, Donner A. Extension of the modified Poisson regression model to prospective studies with correlated binary data. Stat Methods Med Res. 2013 Dec;22(6):661-70.

[10] Greenland S. Model-based estimation of relative risks and other epidemiologic measures in studies of common outcomes and in case-control studies. Am J Epidemiol. 2004 Aug 15;160(4):301-5.

[11] Yelland LN, Salter AB, Ryan P. Performance of the modified Poisson regression approach for estimating relative risks from clustered prospective data. Am J Epidemiol. 2011 Oct 15;174(8):984-92.

[12] Chen W, Qian L, Shi J, Franklin M. Comparing performance between log-binomial and robust Poisson regression models for estimating risk ratios under model misspecification. BMC Med Res Methodol. 2018 Jun 22;18(1):63.

[13] Keppel, G., & Wickens, T. D. Design and analysis: A researcher's handbook (4th ed.). Pearson Prentice Hall; 2004.

[14] Maxwell, S. E., & Delaney, H. D. Designing experiments and analyzing data: A model comparison perspective (2nd ed.). Lawrence Erlbaum Associates; 2004.

[15] Schuemie MJ, Hripcsak G, Ryan PB, Madigan D, Suchard MA. Empirical confidence interval calibration for population-level effect estimation studies in observational healthcare data. Proceedings of the National Academy of Sciences. 2018;115(11):2571–7.

[16] Schuemie MJ, Ryan PB, DuMouchel W, Suchard MA, Madigan D. Interpreting observational studies: why empirical calibration is needed to correct p-values. Stat Med. 2014;33(2):209–18.

© 2024 Zhou T et al. *JAMA Network Open.*
