# Supplementary material for: Body Mass Index and Postacute Sequelae of SARS-CoV-2 Infection in Children and Young Adults
Source: JAMA Netw Open. 2024 Oct 28;7(10):e2441970. doi: 10.1001/jamanetworkopen.2024.41970 (PMC11581483; doi:10.1001/jamanetworkopen.2024.41970)
Supplement: Supplement 2. — Data Sharing Statement [file jamanetwopen-e2441970-s002.pdf]

Data Sharing Statement

Zhou. Body Mass Index and Postacute Sequelae of SARS-CoV-2 Infection in Children and Young Adults. *JAMA Netw Open*. Published October 28, 2024.
doi:10.1001/jamanetworkopen.2024.41970

Data
Data available: No

Additional Information
Explanation for why data not available: The study is based on a national multi-site cohort study with electronic health records (EHR) data. The request to the data access can be submitted via https://recovercovid.org/.
